# Identifying novel strategies for treating human hair loss disorders: Cyclosporine A suppresses the Wnt inhibitor, SFRP1, in the dermal papilla of human scalp hair follicles

**DOI:** 10.1371/journal.pbio.2003705

**Published:** 2018-05-08

**Authors:** Nathan J. Hawkshaw, Jonathan A. Hardman, Iain S. Haslam, Asim Shahmalak, Amos Gilhar, Xinhong Lim, Ralf Paus

**Affiliations:** 1 Centre for Dermatology Research, University of Manchester, Manchester Academic Health Science Centre and NIHR Manchester Biomedical Research Centre, Manchester, United Kingdom; 2 Department of Biological Sciences, School of Applied Sciences, University of Huddersfield, Huddersfield, United Kingdom; 3 Crown Clinic, Manchester, United Kingdom; 4 Skin Research Laboratory, Faculty of Medicine, Technion - Israel Institute of Technology, Haifa, Israel; 5 Institute of Medical Biology, Agency for Science, Technology, and Research, Singapore; 6 Skin Research Institute of Singapore, Singapore; 7 Lee Kong Chian School of Medicine, Nanyang Technological University, Singapore; 8 Duke-NUS Medical School, Singapore; 9 Department of Dermatology and Cutaneous Surgery, University of Miami Miller School of Medicine, Miami, Florida, United States of America; Stanford University School of Medicine, United States of America

## Abstract

Hair growth disorders often carry a major psychological burden. Therefore, more effective human hair growth–modulatory agents urgently need to be developed. Here, we used the hypertrichosis-inducing immunosuppressant, Cyclosporine A (CsA), as a lead compound to identify new hair growth–promoting molecular targets. Through microarray analysis we identified the Wnt inhibitor, secreted frizzled related protein 1 (SFRP1), as being down-regulated in the dermal papilla (DP) of CsA-treated human scalp hair follicles (HFs) ex vivo. Therefore, we further investigated the function of SFRP1 using a pharmacological approach and found that SFRP1 regulates intrafollicular canonical Wnt/β-catenin activity through inhibition of Wnt ligands in the human hair bulb. Conversely, inhibiting SFRP1 activity through the SFRP1 antagonist, WAY-316606, enhanced hair shaft production, hair shaft keratin expression, and inhibited spontaneous HF regression (catagen) ex vivo. Collectively, these data (a) identify Wnt signalling as a novel, non–immune-inhibitory CsA target; (b) introduce SFRP1 as a physiologically important regulator of canonical β-catenin activity in a human (mini-)organ; and (c) demonstrate WAY-316606 to be a promising new promoter of human hair growth. Since inhibiting SFRP1 only facilitates Wnt signalling through ligands that are already present, this ‘ligand-limited’ therapeutic strategy for promoting human hair growth may circumvent potential oncological risks associated with chronic Wnt over-activation.

## Author summary

Hair loss is a common disorder and can lead to psychological distress. Cyclosporine A, a fungal metabolite commonly used as an immunosuppressant, can potently induce hair growth in humans. However, it cannot be effectively used to restore hair growth because of its toxic profile. In this study, we used Cyclosporine A as a lead compound to identify novel therapeutic targets that can aid the development of new hair growth–promoting agents. Through microarray analysis, we found that the level of the secreted Wnt inhibitor, *SFRP1*, was significantly reduced by Cyclosporine A. This inspired us to design a new pharmacological approach that uses WAY-316606, a reportedly well-tolerated and specific antagonist of SFRP1, to prolong the growth phase of the hair cycle. We show that WAY-316606 enhances human hair growth ex vivo, suggesting that it is a more targeted hair growth promoter with the potential to treat human hair loss disorders.

## Introduction

The current pharmacological treatment for hair loss disorders is unsatisfactory, with patients being limited to only two FDA-approved hair growth promoters (minoxidil and finasteride), neither of which is robustly and universally efficacious [1]. Given the severe psychological burden and negative quality of life that can be associated with hair loss, additional, but safe, human hair growth–promoting agents are urgently needed.

There are relatively few known drugs that cause excessive hair growth (hypertrichosis) in patients. Among these, the immunosuppressive calcineurin inhibitor, Cyclosporine A (CsA), most frequently and characteristically induces hypertrichosis [2,3]. CsA also prolongs active hair growth (anagen) in organ-cultured human scalp hair follicles (HFs) ex vivo [4,5]. Likely, the hair growth–stimulatory effects of CsA are independent of its T cell–inhibitory activity, because human HFs grafted onto immunocompromised nude mice treated with CsA also show anagen prolongation in vivo [6].

CsA effects on hair growth have been studied most extensively in mice (S1 and S2 Tables), in which CsA induces anagen in quiescent (telogen) HFs [7], reportedly through blocking the nuclear translocation of nuclear factor of activated T cells (NFATc)1 in epithelial HF stem cells (eHFSCs) [8]. Conversely, CsA inhibits HF regression (catagen) [9] (S2 Table), presumably through blocking NFATc2 within the murine hair matrix [10]. However, this does not satisfactorily explain how CsA prolongs anagen in human scalp HFs, because neither active NFATc1 nor NFATc2 protein are expressed within the human anagen HF bulb [5], i.e., where catagen development is initiated [1]. Therefore, we hypothesised that other molecular pathways must underlie the anagen-prolonging activity of CsA in human HFs [5], and that modulating these novel targets could achieve hair growth with better efficacy and fewer side effects than CsA itself.

To test these hypotheses, we treated organ-cultured anagen VI human scalp HFs [11] with a therapeutic dose of CsA (10^−7^M) [5], followed by microarray analysis, in situ hybridisation (ISH), quantitative real-time PCR (qRT-PCR), immunofluorescence microscopy, and pharmacological assays. To eliminate any potentially confounding effects on bulge eHFSCs [8], and given that eHFSCs are not actively involved in mediating the anagen–catagen transition [12], we exclusively focused on the effects of CsA within the human anagen hair bulb. Following these experiments we identified the endogenous Wnt inhibitor, secreted frizzled related protein 1 (SFRP1), as a novel target of CsA treatment. SFRP1 activity can be directly targeted with the antagonist, WAY-316606, which enhances human hair growth ex vivo.

## Results and discussion

**Fig 1:**
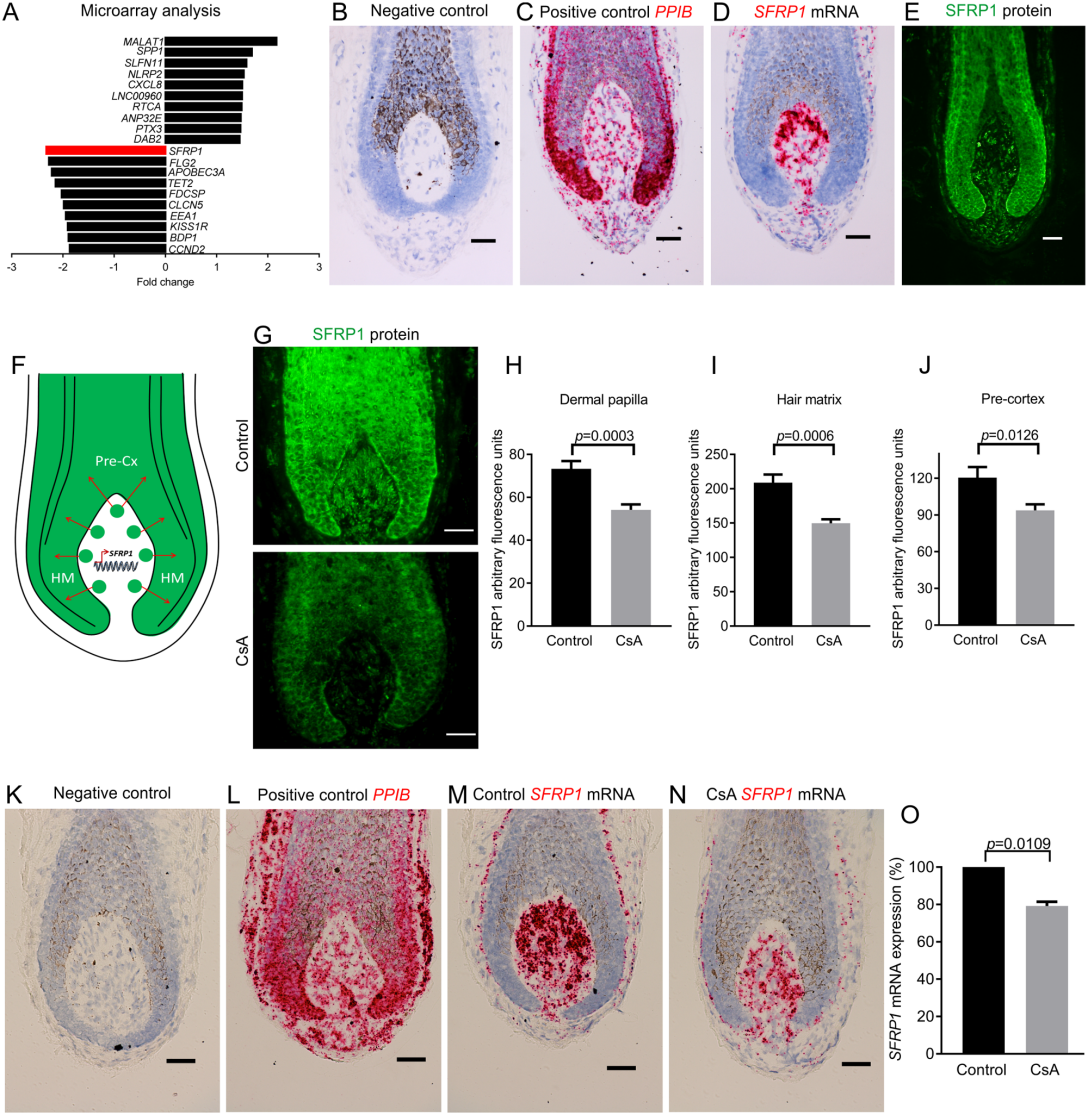
CsA treatment down-regulates SFRP1 in the DP of human HFs ex vivo. (**A**) Microarray analysis of CsA-treated (6 hours) human HFs (*n* = 3 male patient samples). (**B–E**) Characterisation of *SFRP1* mRNA (in situ hybridisation; **B–D**) and protein (immunofluorescence; **E**) expression in the human HF bulb. (**F**) Schematic of SFRP1 in the human HF bulb. (**G–J**) SFRP1 protein analysis after CsA treatment (48 hours) in the human HF bulb (*n* = 10 HFs Control, 11 HFs CsA; from 2 male patient samples). **(K–N)** In situ hybridisation of *SFRP1* mRNA with CsA treatment (48 hours). (**O**) qRT-PCR analysis of *SFRP1* after CsA treatment (48 hours) (*n* = 3 male patient samples). (H and J) Two-tailed unpaired *t* test, (I) Mann-Whitney test, and (O) one-sample *t* test. Data are expressed as mean ± SEM. Scale bars = 50 μm. Underlying data can be found in S1 Data. *ANP32E*, acidic nuclear phosphoprotein 32 family member E; *APOBEC3A*, apolipoprotein B mRNA editing enzyme catalytic subunit 3A; *BDP1*, B double prime 1, subunit of RNA polymerase III transcription initiation factor IIIB; *CCND2*, cyclin D2; CsA, Cyclosporine A; *CLCN5*, chloride voltage-gated channel 5; *CXCL8*, C-X-C motif chemokine ligand 8; *DAB2*, DAB2, clathrin adaptor protein; DP, dermal papilla; *EEA1*, early endosome antigen 1; *FDCSP*, follicular dendritic cell secreted protein; *FLG2*, filaggrin family member 2; HF, hair follicle; HM, hair matrix; *KISS1R*, KISS1 receptor; *MALAT1*, metastasis associated lung adenocarcinoma transcript 1; *NLRP2*, NLR family pyrin domain containing 2; Pre-Cx, pre-cortex; qRT-PCR, quantitative real-time PCR; *PPIB*, peptidylprolyl isomerase B; *PTX3*, pentraxin 3; *RTCA*, RNA 3′-terminal phosphate cyclase; SFRP1, secreted frizzled related protein 1; *SLFN11*, schlafen family member 11; *SPP1*, secreted phosphoprotein 1; *TET2*, tet methylcytosine dioxygenase 2.

To identify novel CsA targets, we treated human HFs with CsA ex vivo (6 hours) and measured primary changes in gene transcription through microarray analysis. Among many other differentially regulated genes (S1 Fig), the Wnt inhibitor, SFRP1 [13], demonstrated the largest decrease (−2.3 fold change, *p* = 4.90E−05) (Fig 1A). As SFRP1 has not previously been investigated in either the CsA literature or in the human hair bulb (S1 and S3 Tables), we next investigated which intrafollicular cell population is responsible for the CsA-induced change in *SFRP1* transcription. Using ISH, we found *SFRP1* mRNA to be exclusively transcribed by fibroblasts associated with the human HF bulb, with the highest *SFRP1* expression seen within the dermal papilla (DP) (Fig 1B–1D and S2 Fig).

Next, we wanted to determine if SFRP1 protein is restricted to or secreted from the DP. Immunofluorescence microscopy revealed that SFRP1 protein can be detected in both the DP and the adjacent epithelial hair matrix and pre-cortex of human scalp HFs (Fig 1E and S2 Fig). Based on the distinct expression patterns of *SFRP1* mRNA (DP) and protein (DP and hair matrix), these data suggest that SFRP1 is transcribed and translated in the DP and is then secreted into the hair matrix and pre-cortex (Fig 1F).

We next asked by quantitative immunohistomorphometry whether CsA inhibits SFRP1 protein production in the human anagen hair bulb. This revealed a significant decrease in SFRP1 protein expression within the DP (Fig 1G and 1H) and in the surrounding HF epithelium (matrix and pre-cortex) (Fig 1G, 1I and 1J) after 48 hours of CsA treatment. Therefore, CsA suppresses SFRP1 protein levels within the human HF bulb. This was corroborated by ISH, which demonstrated a decrease in *SFRP1* mRNA within the DP after CsA treatment (Fig 1K–1N), and by qRT-PCR, which showed a significant decrease in intrafollicular *SFRP1* mRNA after 48 hours (Fig 1O).

To determine if CsA’s hair growth–promoting effects occur through SFRP1 and if SFRP1 negatively impacts human hair growth, we first treated human HFs ex vivo with CsA and vehicle control. As a positive control, we confirmed that the percentage of anagen VI HFs was significantly higher after CsA treatment compared to vehicle control on days 4 and 6 (S4A–S4C Fig), validating previous reports that CsA prolongs the duration of anagen [4,5] (S2 Table). Next, HFs were treated with vehicle control, recombinant human SFRP1 (rhSFRP1) alone, and rhSFRP1 together with CsA. Macroscopically, the treatment of rhSFRP1 induced more HFs to prematurely enter catagen by day 4 compared to the vehicle control (S4D–S4G Fig). The ability of CsA to prolong anagen (S4A Fig) was blocked by the addition of rhSFRP1 with CsA, as the percentage of anagen HFs was comparable to vehicle control over 6 days in culture (S4D Fig). Next, we analysed morphological changes with vehicle control, rhSFRP1 alone, and rhSFRP1 with CsA-treated HFs at day 6 using Ki-67/TUNEL immunofluorescence (S5A–S5C and S6 Figs). Surprisingly, the percentage of Ki-67+ cells below Auber’s line was highest with rhSFRP1 treatment (S5E Fig). However, this did not functionally translate into hair growth stimulation, as rhSFRP1 alone drastically reduced hair matrix size (S5D Fig), whereas treatment with rhSFRP1 and CsA together showed no significant difference in hair matrix size compared to vehicle control (S5D Fig). Treatment with rhSFRP1 enhanced the emigration of DP fibroblasts to the connective tissue sheath (S5H Fig), which occurs upon catagen induction, leading to a reduced DP volume [14,15]. Also, rhSFRP1 promoted apoptosis in fibroblasts emigrating from the DP, which is indicative of catagen induction (S5I Fig) [14,15]. Conversely, there was no difference between control and rhSFRP1 with CsA in the emigration of fibroblasts (S5H Fig) or TUNEL+ cells in the DP stalk (S5I Fig). Collectively, this (a) demonstrates that enhanced SFRP1 activity induces premature catagen and (b) suggests that CsA hair growth–promoting effects occur at least in part through inhibiting SFRP1, since the addition of rhSFRP1 prevented CsA treatment from maintaining anagen and modulating many of these parameters above vehicle control baseline.

Therefore, irrespective of any effects CsA might also exert on human eHFSCs, CsA treatment can target the DP of human scalp HFs, i.e., the mesenchymal inductive control centre [12,16]. This important mesenchymal HF target had been unrecognised in previous murine work (S1 Table) yet is in line with our previous finding that CsA inhibits the emigration of fibroblasts from the DP in human HFs [5]. This also demonstrates that CsA suppresses a mesenchymal-to-epithelial signal via SFRP1.

**Fig 2:**
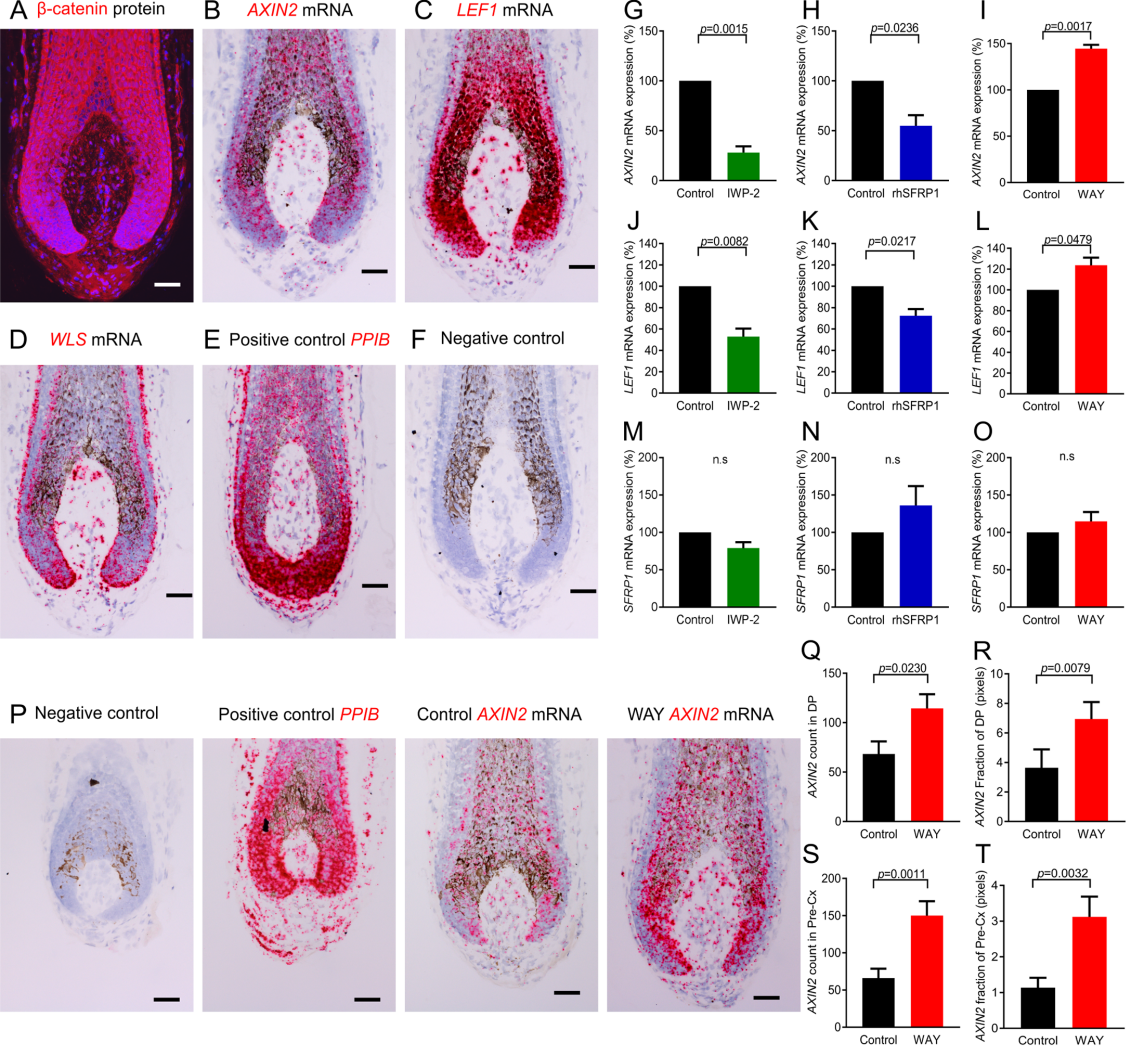
SFRP1 modulates canonical β-catenin activity at the ligand level in the human HF bulb ex vivo. **(A–F)** The human HF bulb expresses the core components of the canonical β-catenin pathway. Immunofluorescence of β-catenin (**A**), in situ hybridisation of *AXIN2* (**B**), *LEF1* (**C**), and *WLS* (**D**). **(G–O)** qRT-PCR analysis of *SFRP1* and the direct β-catenin target genes *AXIN2* and *LEF1* in the human HF with IWP-2 (**G, J, M**), rhSFRP1 (**H, K, N**), and WAY-316606 (**I, L, O**) treatment (*n* = 4 male patient samples). (**P–T**) Analysis of *AXIN2* mRNA by in situ hybridisation after WAY-316606 treatment (*n* = 15 HFs control, 14 HFs WAY-316606; from 3 male patient samples). (G–O) One-sample *t* test, (Q and S) two-tailed unpaired *t* test (R and T), and Mann-Whitney test. Data are expressed as mean ± SEM. Scale bars = 50 μm. Underlying data can be found in S1 Data. *AXIN2*, axis inhibition protein 2; DP, dermal papilla; HF, hair follicle; IWP-2, inhibitor of Wnt production-2; *LEF1*, lymphoid enhancer binding factor 1; n.s, not significant; *PPIB*, peptidylprolyl isomerase B; Pre-Cx, pre-cortex, qRT-PCR, quantitative real-time PCR; rhSFRP1, recombinant human SFRP1; SFRP1, secreted frizzled related protein 1; WAY, WAY-316606; *WLS*, Wntless.

Given the key role of SFRP1 as an inhibitor of canonical Wnt signalling [17], we next asked whether Wnt ligand activity modulates canonical β-catenin in the human HF bulb. While Wnt ligand activity maintains anagen and hair shaft formation in the murine HF [18,19], it is unclear whether this directly applies to humans. The distribution of cells that are Wnt active has been described in the human HF bulb [20,21]. However, there are clear discrepancies with the reported nuclear localisation of both β-catenin and the key canonical transcription factor lymphoid enhancer binding factor 1 (LEF1) in the pre-cortex [21,22] and the DP [20,23]. Therefore, we wanted to identify which cells are precisely active for canonical β-catenin signalling. Immunofluorescence microscopy showed nuclear localisation of β-catenin in both selected human HF fibroblasts (DP and DP stalk cells) and epithelial cells (hair matrix and pre-cortical hair matrix keratinocytes) of human scalp HFs (Fig 2A and S7 Fig). In addition, ISH revealed that the direct canonical β-catenin target genes axis inhibition protein 2 (*AXIN2*) and *LEF1* are both transcribed in these cell populations (Fig 2B and 2C). This identifies that Wnt/β-catenin signalling is active in both epithelial and mesenchymal cells. Importantly, this also demonstrates that β-catenin activity in the epithelial regions is not restricted to the pre-cortex, as previously thought [21], and also occurs in the proliferating hair matrix.

We then asked which cells are secreting Wnt, beginning with analysing the expression of Wntless (*WLS*), which is required for Wnt ligand secretion [18], followed by a panel of Wnt ligands. ISH showed that *WLS* is present throughout the HF bulb, although with varying levels of expression (Fig 2D and S8 Fig). Conversely, among the seven Wnt ligands we probed for, *WNT3*, *WNT4*, *WNT10A*, and *WNT10B* were expressed and restricted to the human epithelial hair matrix and pre-cortex, while *WNT1*, *WNT2*, and *WNT3A* were undetectable (S9 Fig). Next, we assessed whether Wnt ligand activity modulates the canonical β-catenin pathway in the human HF bulb by treating human HFs for 48 hours with inhibitor of Wnt production-2 (IWP-2), a small molecule inhibitor of Wnt ligand secretion [24]. This significantly reduced the transcription of both *AXIN2* and *LEF1* (Fig 2G and 2J). Therefore, Wnt secretion is required for maintaining canonical β-catenin activity in human scalp HFs, perfectly in line with the most recent demonstration that WNT10A is required for normal human skin appendage function [25].

These important background data define the location of key molecular players within the concert of Wnt ligands, β-catenin activity, and SFRP1 production in the human anagen HF. Notably, the secreted SFRP1 protein from the DP overlaps with specific Wnt ligands expressed in the immediately adjacent HF epithelium (S9 Fig), suggesting that SFRP1 may inhibit canonical β-catenin activity. This hypothesis was tested by treating human HFs ex vivo with rhSFRP1. After 48 hours, both *AXIN2* and *LEF1* transcription were significantly reduced (Fig 2H and 2K). Conversely, when we incubated human HFs with WAY-316606, a specific and reportedly well-tolerated antagonist of SFRP1 [26], both *AXIN2* and *LEF1* transcription were significantly increased (Fig 2I and 2L). Therefore, SFRP1 functions as an inhibitor of canonical Wnt/β-catenin signalling in the human hair bulb.

Furthermore, ISH showed a significant increase in *AXIN2* mRNA levels in situ after WAY-316606 treatment within both the pre-cortex and DP (Fig 2P–2T). This demonstrates that despite the presence of spatially very well-defined Wnt ligands (S9 Fig), the general presence of secreted SFRP1 protein within the epithelium serves to moderate Wnt activity as *AXIN2* levels become significantly up-regulated after SFRP1 inhibition by WAY-316606, while still retaining its spatial pattern within the human HF bulb (Fig 2P–2T). In addition, this increase in Wnt activity is not due to an increase in ectopic nuclear β-catenin, as anagen HFs already express maximally high levels of nuclear β-catenin (S10 Fig). Therefore, the intrafollicular increase in β-catenin activity after SFRP1 suppression is due to a response of both pre-cortical HF keratinocytes and DP fibroblasts (both regions are Wnt active, i.e., β-catenin+ *LEF1*+ *AXIN2+*). Notably, *SFRP1* transcription itself did not change after treatment with either IWP-2, rhSFRP1, or WAY-316606 (Fig 2M–2O). This suggests that SFRP1 does not negatively regulate its own expression and indicates that Wnt ligand activity does not control *SFRP1* expression.

Taken together, our data introduce SFRP1 as a physiologically important regulator of canonical β-catenin activity in a human (mini-)organ, the HF. We also present the first evidence that the SFRP1 inhibitor, WAY-316606, effectively enhances β-catenin activity in mammalian skin cell populations, namely in both human hair pre-cortex keratinocytes and DP fibroblasts ex vivo.

β-catenin signalling is essential for murine hair growth through both the anagen-inducing properties of the DP [19,27] and differentiation of hair matrix keratinocytes into hair shaft trichocytes [28,29]. Therefore, we next addressed the clinically crucial question of whether stimulating canonical β-catenin activity within both human HF compartments (Fig 2P–2T) enhances human hair growth.

**Fig 3:**
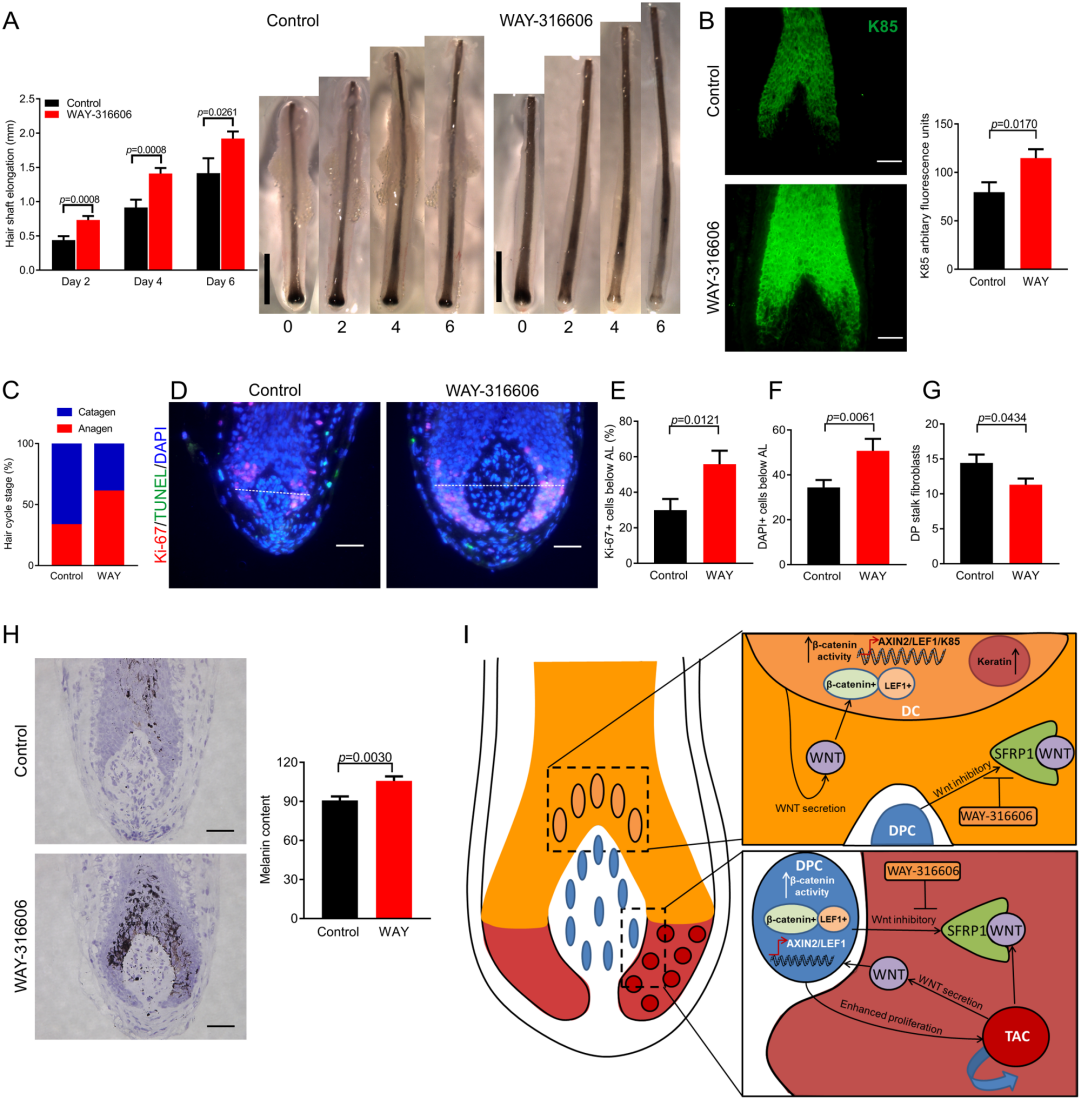
Inhibiting SFRP1 activity with WAY-316606 enhances human hair growth, increases K85 protein expression, and inhibits spontaneous catagen ex vivo. **(A)** Hair shaft elongation of human HFs ex vivo with WAY-316606 treatment (*n* = 31 HFs Control, 30 HFs WAY-316606; from 3 male patient samples). (**B**) K85 protein quantification using immunofluorescence after 48 hours of treatment with WAY-316606 (*n* = 16 HFs control, 14 HFs WAY-316606; from 3 male patient samples). (**C**) Macroscopic quantification of hair cycle stage on day 6 with WAY-316606 treatment. (**D–H**) Validation of HF cycle stage with Ki-67/TUNEL analysis (*n* = 21 HFs control, 20 HFs WAY-316606; from 3 male patient samples) and Masson's-Fontana (*n* = 17 HFs control, 20 HFs WAY-316606; from 3 male patient samples). **(I)** Working hypothesis. Data are expressed as mean ± SEM. (A, B, E, G, and H) Two-tailed unpaired *t* test and (F) Mann-Whitney test. Scale bars, A = 1 mm; B, D, and H = 50 μm. Underlying data can be found in S1 Data. AL, Auber’s line; Axin2, axis inhibition protein 2; CsA, Cyclosporine A; DC, differentiating cell; DP, dermal papilla; DPC, dermal papilla cell; HF, hair follicle; K85, Keratin 85; LEF1, lymphoid enhancer binding factor 1; SFRP1, secreted frizzled related protein 1; TAC, transient amplifying cell; WAY, WAY-316606.

To answer this, human HFs were treated ex vivo with WAY-316606 for 6 days, and hair shaft production was measured. WAY-316606 significantly increased hair shaft production (elongation) as early as 2 days following treatment (Fig 3A), even faster than CsA-induced hair shaft elongation ex vivo, which occurs several days later [4,5]. As canonical β-catenin signalling appears to control the expression of hair keratins necessary for hair shaft production [21,28,29], we also investigated whether WAY-316606 treatment can also impact on human hair keratin expression. Quantitative immunohistomorphometry showed that WAY-316606 treatment rapidly and significantly up-regulated protein expression of the hair shaft keratin K85 (Fig 3B), which is expressed within the pre-cortical hair matrix and the hair shaft [30]. Therefore, antagonising SFRP1 activity enhances human hair shaft production, and do so more effectively than CsA.

Clinically, the most important challenge in effective hair loss management is to prolong the duration of anagen [1]. CsA achieves this through blocking catagen within both mice and man [5,6,9]. It was therefore crucial to determine if antagonising SFRP1 activity through WAY-316606 treatment replicates this effect of CsA.

Indeed, after 6 days of treatment with WAY-316606, a greater percentage of organ-cultured anagen scalp HFs had remained in anagen VI, both macroscopically (Fig 3C and S11 Fig) and microscopically, as validated by standardised, quantitative hair cycle histomorphometry [15] (Fig 3D–3H). This corresponded to a significantly higher percentage of proliferating (Ki-67+) hair matrix keratinocytes (Fig 3D and 3E), a greater number of DAPI+ cells below Auber’s line (Fig 3D and 3F), and a significantly higher melanin content of WAY-316606–treated HFs (Fig 3H). In addition, the number of fibroblasts emigrating from the DP ex vivo was reduced (Fig 3D and 3G), exactly mimicking the effects of CsA [5]. However, there did not appear to be any significant changes in cell death, as analysed by TUNEL staining of the hair matrix, DP, or DP stalk (S12 Fig). Therefore, directly inhibiting SFRP1 activity with WAY-316606 not only stimulates intrafollicular Wnt signalling but maintains anagen human HFs within the maximally proliferative stage of the HF cycle.

Collectively, our study (a) identifies Wnt signalling as a novel target of CsA treatment that appears unrelated to its immunosuppressive activities; (b) introduces SFRP1 as a physiologically important regulator of canonical β-catenin activity in a human (mini-)organ, the HF, by showing that this Wnt inhibitor is a key partner in a potent new mesenchymal-epithelial interaction; and (c) demonstrates that WAY-316606 is a promising new pharmacological promoter of human hair growth, whose toxicity profile [26] is expected to be more favourable than that of CsA. While we have demonstrated that CsA can modulate SFRP1, it cannot be excluded that CsA has additional effects on the immune system or other cell types and may thus indirectly affect catagen entry.

Interestingly, no hair abnormalities have been reported in *Sfrp1* knockout mice. Once again, this cautions against extrapolating from murine to human HF biology and only underscores the clinical importance of studying SFRP1 functions directly in human tissue. However, investigators interested in murine SFRP1 biology might wish to probe whether some of the other abnormalities reported in *Sfrp1* knockout mice, e.g., increased mammary ductal branching [31] or dysregulated glucose metabolism [32], are pharmacologically reproducible in wild-type mice by SFRP1 inhibitor treatment.

Remarkably, the extensive hair CsA literature had missed canonical Wnt signalling as a clinically important target of this widely administered immunosuppressant. The novel CsA-SFRP1-Wnt connection elucidated here may also help to better explain previously ill-understood adverse effects of CsA therapy, namely gingival hyperplasia [33]. The next key challenge is to dissect the as yet unknown molecular mechanisms by which CsA inhibits *SFRP1* transcription (to clarify this was beyond the scope and reach of the current translational study).

The underlying intrafollicular signalling is depicted in Fig 3I: the secreted Wnt inhibitor, SFRP1, controls human HF cycling and hair shaft production after secretion from the DP by interacting with Wnt ligands in the immediately adjacent HF epithelium. Manipulating this novel mesenchymal-epithelial signal with the SFRP1 inhibitor, WAY-316606, increases canonical β-catenin activity in both the DP and the pre-cortex. This boost in Wnt signalling enhances human hair shaft formation and inhibits catagen entry. Therefore, Dickkopf-related protein 1 (DKK1) is not the only important regulator of Wnt activity in the human HF [34], and SFRP1 is a viable alternative target molecule for the therapeutic up-regulation of hair growth–promoting Wnt signalling.

Since CsA binds and inhibits multiple targets (e.g., cyclophilin and calcineurin) [35], which is thought to underlie its serious toxicity profile [3,33], topical inhibition of SFRP1 using WAY-316606 would be a much more targeted approach for stimulating human hair growth without having to use a potent immunosuppressant, particularly as thus far there are no known off-target effects from WAY-316606 treatment. In addition, WAY-316606 is highly selective against other closely related SFRP family members (SFRP2 and SFRP5). For example, at 2 μM, WAY-316606 inhibits SFRP1 activity by about 40%, whereas SFRP2 and SFRP5 activity is only inhibited by about 5% and about 2%, respectively [26]. Moreover, this Wnt disinhibition technique may be a safer long-term therapeutic strategy for stimulating β-catenin activity in the human HF. Because inhibiting SFRP1 by WAY-316606 only facilitates Wnt signalling through ligands that are already present in the human HF, this ‘ligand-limited’ strategy for promoting human hair growth may circumvent potential oncological risks typically associated with β-catenin stabilisation [36].

## Methods

### Ethics statement

Tissue samples were harvested with written informed patient consent and with approval from the Manchester Skin Health Biobank (UK North West—Haydock Research Ethics Committee approved study 14/NW/0185).

### Human HF organ culture

Male occipital scalp HFs were obtained from patients undergoing hair transplant surgery at the Crown Cosma Clinic, Manchester, UK. Tissue was dissected, with individual full-length anagen VI HFs cultured in serum-free Williams’ E medium (Gibco, Paisley, UK) supplemented with 2 mM L-glutamine (Invitrogen, Paisley, UK), 10 ng/mL hydrocortisone (Sigma, Dorset, UK), and 1% antibiotic/antimycototic mixture (100×, Gibco) and incubated overnight at 37 °C and 5% CO_2_ as described [5,11]. The following day, HFs that had macroscopically remained in anagen VI had media replaced with the following additions:

#### CsA

HFs were cultured with 500 μL of CsA at 10^−7^ M (Cat no. #9973S, Cell Signalling Technology, Beverly, MA) or vehicle control. CsA solutions (both stock [10^−3^M] and working [10^−7^M]) and vehicle controls were made as previously described [4]. For microarray analysis, HFs were incubated for 6 hours with CsA, while immunofluorescent, ISH, and qRT-PCR validation occurred at 48 hours incubation.

#### WAY-316606

HFs were cultured with 500 μL of WAY-316606 at 2 μM (Cat no. A3932, ApexBio; Houston, TX) or vehicle control (0.02% DMSO). A stock solution of WAY-316606 was made at a 10 mM concentration by adding 2,229.8 μL of DMSO (Sigma) to 10 mg of WAY-316606. This was subsequently diluted in serum-free HF media (4 μL of WAY-316606 10 mM in 19,996 μL HF media) for a working 2 μM concentration. For qRT-PCR analysis, HFs were incubated with WAY-316606 for 24 hours. To analyse β-catenin activity (ISH [*AXIN2* mRNA] and immunofluorescent analysis [nuclear β-catenin]), HFs were incubated for 48 hours with WAY-316606. For hair cycle analysis, HFs were incubated with WAY-316606 up to 6 days.

#### Carrier-free rhSFRP1

HFs were cultured with 1 mL carrier-free rhSFRP1 at 10 μg/mL (Cat No. 5396-SF-025, R&D Systems; Minneapolis, MN) or vehicle control (PBS). A stock solution of rhSFRP1 was made at 250 μg/mL by adding 100 μL of PBS (Gibco) to 25 μg of rhSFRP1. This was subsequently diluted in serum-free HF media (40 μL of rhSFRP1 [250 μg/mL] in 960 μL HF media) for a working concentration of 10 μg/mL. For qRT-PCR analysis, HFs were incubated with rhSFRP1 for 48 hours.

#### CsA and rhSFRP1 rescue experiment

Stock solutions were made as described above and subsequently diluted further to the following final solutions: vehicle control (960 μL of CsA vehicle [containing ethanol, TWEEN-80 in serum-free HF media] and 40 μL of PBS), rhSFRP1 at 10 μg/mL (960 μL of CsA vehicle [containing ethanol, TWEEN-80 in serum-free HF media] and 40 μL rhSFRP1), CsA at 10^−7^ M with rhSFRP1 at 10 μg/mL (960 μL of CsA [containing ethanol, TWEEN-80 in serum-free HF media] and 40 μL rhSFRP1). HFs were then cultured in 500 μL of corresponding solution per well. For hair cycle analysis, HFs were incubated with vehicle control, rhSFRP1 alone, or rhSFRP1 with CsA up to 6 days.

#### IWP-2

HFs were cultured with 500 μL of IWP-2 at 5 μM (Cat No. 04–0034, Stemgent; Lexington, MA) or vehicle control (0.1% DMSO). A stock solution of IWP-2 was made at 5 mM concentration by diluting 857.3 μL of DMSO to 2 mg of IWP-2. This was subsequently diluted in serum-free HF media (10 μL of IWP-2 [5 mM] in 9,990 μL of HF media) for a working concentration of 5 μM. For qRT-PCR analysis, HFs were incubated with IWP-2 for 48 hours.

For 6-day HF organ culture, media was changed every other day and HF images for measuring hair shaft elongation were taken with a Leica EC3 camera (Leica, Wetzlar, Germany). Macroscopic quantification of HF stages were carried out as previously described [11,15,37]. On day 6 following WAY-316606 treatment, studies were terminated with HFs being embedded in optimal cutting temperature compound (OCT [Thermo-scientific, Leicestershire, UK]) and frozen in liquid nitrogen. Samples were stored at −80 °C prior to subsequent use. For qRT-PCR studies, HFs were harvested and stored in 1 mL of RNAlater (Ambion, Paisley, UK) at 4 °C until required.

### Quantitative immunohistomorphometry

Frozen sections from microdissected fresh, 2-, and 6-day cultured HFs were made using a cryostat (OTF5000, Bright, London, UK) at 6 μm. Masson-Fontana staining and Ki-67/TUNEL dual immunofluorescence and histomorphometric analysis was carried out as previously described [15]. Masson-Fontana, Ki-67/TUNEL, SFRP1, and K85 staining were imaged using Biozero 8000 Keyence microscope (Biozero, Osaka, Japan) for image analysis. β-catenin was imaged using the Olympus BX53 upright microscope (Olympus, Tokyo, Japan). Representative images for ISH were imaged using Olympus BX53 upright microscope. ImageJ was used to quantify immunofluorescent intensity and ISH signal. For some representative images, the contrast was changed globally within CorelDraw or PowerPoint, and matching settings were applied to both test and control.

### SFRP1 and β-catenin immunofluorescence

Frozen HF sections were fixed in 4% PFA for 20 minutes at 4 °C, then washed with Tris-buffered saline (TBS [all wash steps used TBS]). Tissue was then permeabilised with 0.5% Triton X-100 (in TBS) for 10 minutes. Sections were washed and incubated with 10% normal goat serum (NGS [in TBS]) for 30 minutes. Next, sections were incubated with either SFRP1 (1:200 in 10% NGS; abcam Cat No. ab4193) or β-catenin (1:200 in 10% NGS; BD Transduction Laboratories Cat No. 610154) primary antibodies overnight (4 °C). The following day, sections were washed and incubated with AF-488 (1:200 in TBS; Invitrogen Cat No. A11008) or AF-594 (1:200 in TBS; Invitrogen Cat No. A11032) secondary antibodies (1 hour at room temperature). Sections were then washed and nuclei were counterstained by using DAPI (1 μg/mL in PBS) for 1 minute. For negative controls, primary antibody raised against SFRP1 and β-catenin were omitted. Frozen human testis was used as a positive control for SFRP1 [38] (S2J and S2K Fig), as well as analysing the differential pattern of SFRP1 mRNA and protein throughout the human HF (S3 Fig).

### K85 immunofluorescence

K85 immunofluorescence was performed on microdissected HFs as per SFRP1 and β-catenin, except that samples were fixed with acetone (10 minutes at −20 °C); no permeabilisation was required and phosphate buffered saline was used for washes. A primary K85 antibody (gift from Dr. Lutz Langbein, Heidelberg, Germany) was used at 1:1,000 concentration (in 2% NGS) and secondary antibody goat anti–guinea pig AF-488 (Invitrogen Cat No. A11073) 1:200 concentration (in 2% NGS). For negative controls, primary antibody raised against K85 was omitted.

### RNA ISH

OCT-embedded HFs were sectioned at 6 μm, dried at −20 °C for 1 hour, and then stored at −80 °C for subsequent use. Sections were then processed for RNA in situ detection using the RNAscope 2.5 HD Reagent Kit-Red (Advanced Cell Diagnostics, Milan, Italy) following manufacturer’s instructions. The following probes were used: SFRP1 (NM_003012.4, target 401–1971), AXIN2 (NM_004655.3, target 502–1674), LEF1 (NM_001166119.1_ target 793–1919), WLS (NM_001193334.1, target 355–1325), WNT1 (NM_005430.3_target 390–1863), WNT2 (NR_024047.1, target 1324–2598), WNT3 (NM_030753.4, target 1014–1945), WNT3A (NM_033131.3, target 1212–2328), WNT4 (NM_030761.4, target 132–1770), WNT10A (NM_025216.2, target 658–1949), WNT10B (NM_003394.3, target 865–2282), PPIB as a positive control (NM_000942.4, region 139–989), DapB as a negative control (EF191515, target 414–862). Quantification of ISH was carried out as previously described [39].

### RNA extraction and qRT-PCR

Total RNA was extracted from 5 full-length HFs for each condition using the RNAeasy mini kit (Qiagen, Manchester, UK) according to manufacturer’s protocol and guidelines. One hundred nanograms of RNA were converted to cDNA using Tetro cDNA synthesis kit (Bioline, London, UK) according to manufacturer’s protocol. Quantitative PCR was performed in triplicate using Taqman probes (Life Technologies, Taqman assay ID: SFRP1: Hs00610060_m1, AXIN2: Hs01063170_m1, LEF1: Hs01547250_m1, GAPDH: Hs02758991_g1). Reactions were performed and analysed using the StepOnePlus Real-Time PCR system and associated software (Applied Biosystems, Paisley, UK). Relative expression was calculated using the ΔCT method against the housekeeping gene, GAPDH.

### Microarray

Human HFs were treated for 6 hours with CsA (10^−7^M) and total RNA was extracted using RNAeasy mini kit. Extracted RNA was taken to the Genomic Core Facilities at the University of Manchester and run onto Affymetrix U133 plus 2.0 GeneChip. Data were deposited in NCBI Geo (GSE109632).

### Bioinformatics analysis

Technical quality control and outlier analysis were performed with dChip (V2005) using the default settings. Background correction, quantile normalisation, and gene expression analysis were performed using RMA in Bioconductor. To establish relationships and compare variability between samples, principal components analysis (PCA) was used, because this method is able to reduce the effective dimensionality of complex gene-expression space without significant loss of information. Differential expression analysis was performed with Limma using a paired test and functions lmFit and eBayes. Gene lists of differentially expressed genes were controlled for false discovery rate (fdr) errors using the method of QVALUE.

### Ingenuity pathway analysis

The microarray data set was uploaded to ingenuity pathway analysis (IPA) and filtered with a *p*-value of 0.05 and examined through core pathway analysis.

### Statistics

All experiments used HFs from at least three individual male patient samples, unless stated otherwise.

Results were first checked for normal distribution using D’Agostino-Pearson omnibus normality test and equal variance using *F*-test. Then, depending on these results, statistical analysis was performed with suitable *t* tests, either parametric (unpaired *t* test) or nonparametric tests (unpaired Mann-Whitney test). For experiments with more than one group, unpaired one-way ANOVA was chosen for normally distributed data with Tukey test to correct for multiple comparisons, while unpaired Kruskal-Wallis test was selected for nonparametric data with Dunn’s test to correct for multiple comparisons. For qRT-PCR analysis, one-sample *t* test was used, with a hypothetical value set to 100 for normalised control. *P*-values <0.05 were considered significant. All statistical analysis was carried out in GraphPad Prism version 7.

## Supporting information

S1 Fig IPA of microarray data from CsA-treated human hair follicles ex vivo. IPA identifies numerous canonical pathways that change with CsA treatment. IPA identifies a significant number of genes changed with CsA treatment that are known to be regulated by certain upstream regulators, predicting whether they are either activated or inhibited. Underlying data can be found in S1 Data. CsA, Cyclosporine A; ERK5, extracellular signal regulated kinase 5; ESR1, estrogen receptor 1; HGF, hepatocyte growth factor; IPA, ingenuity pathway analysis; TGFB1, transforming growth factor beta 1.
(TIF)

S2 Fig Characterisation of SFRP1 mRNA and protein in the human HF bulb. (**A–C**) Using in situ hybridisation, *SFRP1* mRNA can be detected in fibroblast populations within the human HF bulb. (**A**) *SFRP1* mRNA is localised to the DP, (**B**) CTS, and (**C**) DP stalk. (**D–G**) SFRP1 protein can also be visualised in the same population of cells using immunofluorescence. (**H**) SFRP1 protein is also detected in the adjacent epithelial regions. (**I**) Diagram of SFRP1 mRNA and protein in the human HF bulb. (**J** and **K**) Human testis was used as a positive control for SFRP1 immunofluorescence. Scale bars, **A**, **D**, and **J** = 50 μm; **B**, **C**, **F**, and **K** = 20 μm; **E**, **G**, and **H** = 10 μm. CTS, connective tissue sheath; DP, dermal papilla; HF, hair follicle; HM, hair matrix; Pre-Cx, pre-cortex; SFRP1, secreted frizzled related protein 1.
(TIF)

S3 Fig SFRP1 mRNA and protein throughout the human hair follicle. (**A–D**) negative control for ISH, (**E–H**) positive control (*PPIB mRNA)* for ISH, (**I–L**) *SFRP1* mRNA, and (**M–P**) SFRP1 protein. Scale bars, **A–L** = 30 μm and **M–P** = 20 μm. ISH, in situ hybridisation; *PPIB*, peptidylprolyl isomerase B; SFRP1, secreted frizzled related protein 1.
(TIF)

S4 Fig CsA anagen prolongation is inhibited by the addition of rhSFRP1. (**A**) Quantification of hair cycle stage with human HFs (ex vivo) treated with vehicle control and CsA (*n* = 8–18 HFs per group; from 4 male patient samples). Macroscopic examples of (**B**) vehicle control HFs and (**C**) CsA-treated HFs after 6 days in culture. **(D)** Quantification of hair cycle stage with human HFs (ex vivo) treated with vehicle control, rhSFRP1 alone, and rhSFRP1 with CsA over 6 days (*n* = 18 HFs per group; from 3 male patient samples). (**E**) Macroscopic examples of vehicle control HFs, (**F**) rhSFRP1-treated HFs, and (**G**) rhSFRP1 plus CsA-treated HFs at day 4. Data are expressed as mean ± SEM; (A) two-tailed unpaired *t* test; *p < 0.05 and **p < 0.01. Scale bars = 1 mm. Underlying data can be found in S1 Data. CsA, Cyclosporine A; HF, hair follicle; rhSFRP1, recombinant human SFRP1.
(TIF)

S5 Fig Ki-67/TUNEL analysis of human HFs treated with rhSFRP1 alone or rhSFRP1 with CsA. Human HFs were treated with vehicle control (**A**), rhSFRP1 only (**B**), or rhSFRP1 with CsA (**C**) for 6 days and subjected to Ki-67/TUNEL analysis (**D–I**) (*n* = 12–15 HFs per group; from 3 male patient samples). **D** and **H** = one-way ANOVA; **E**, **F**, **G**, and **I** = Kruskal-Wallis test; data are expressed as mean ± SEM; dotted white line depicts Auber’s line; scale bars = 50 μm. Underlying data can be found in S1 Data. CsA, Cyclosporine A; HF, hair follicle; rhSFRP1, recombinant human SFRP1.
(TIF)

S6 Fig Ki-67/TUNEL images of human HFs treated with rhSFRP1 alone or rhSFRP1 with CsA. Human HFs were treated with vehicle control (**A**), rhSFRP1 only (**B**), or rhSFRP1 with CsA (**C**) for 6 days and subjected to Ki-67/TUNEL analysis. Dotted white line depicts Auber’s line; scale bars = 30 μm. CsA, Cyclosporine A; HF, hair follicle; rhSFRP1, recombinant human SFRP1.
(TIF)

S7 Fig Characterisation of β-catenin in the human hair follicle bulb. **(A–E)** Using immunofluorescence, active (nuclear) β-catenin can be detected throughout the human hair follicle bulb, (**B**) pre-cortex, (**C**) dermal papilla, (**D**) hair matrix, and (**E**) dermal papilla stalk. White arrows depict nuclear β-catenin. Dashed white lines highlight regions of interest. Scale bars, **A** = 50 μm; **B–E** = 20 μm. DP, dermal papilla; HM, hair matrix; Pre-Cx, pre-cortex.
(TIF)

S8 Fig Characterisation of *WLS* mRNA in the human hair follicle bulb. Using in situ hybridisation, *WLS* mRNA can be detected in both epithelial (**B–D**) and mesenchymal (**E–G**) cell populations within the human hair follicle bulb. Dashed yellow lines highlight regions of interest. Scale bars, **A** = 50 μm, **B–G** = 20 μm. CTS, connective tissue sheath; DP, dermal papilla; HM, hair matrix; IRS, inner root sheath; Pre-Cx, pre-cortex; *WLS*, Wntless.
(TIF)

S9 Fig Characterisation of Wnt ligand mRNA in the human hair follicle bulb. Using ISH, the Wnt ligands *WNT3* (**A**), *WNT4* (**B**), *WNT10A* (**C**), and *WNT10B* (**D**) can be detected in epithelial cells of the human hair follicle bulb, whereas *WNT1* (**E**), *WNT2* (**F**), and *WNT3A* (**G**) were not detected. (**H**) Negative control and (**I**) positive control for ISH. (**J**) Schematic of the Wnt ligands *WNT3*, *WNT4*, *WNT10A*, and *WNT10B*. Scale bars = 50 μm. CTS, connective tissue sheath; DP, dermal papilla; HM, hair matrix; IRS, inner root sheath; ISH, in situ hybridisation; mRNA, messenger ribonucleic acid; ORS, outer root sheath.
(TIF)

S10 Fig Nuclear β-catenin quantification with WAY-316606 treatment in the human hair follicle bulb ex vivo. **(A–C)** Nuclear β-catenin quantification using immunofluorescence within the DP after WAY-316606 treatment (48 hours). **(D–F)** Nuclear β-catenin quantification using immunofluorescence within the Pre-Cx after WAY-316606 treatment (48 hours) (*n* = 13 HFs control, 14 HFs WAY-316606; from 3 male patient samples). Data are expressed as mean ± SEM. Dotted lines depict regions analysed, DP (**A** and **B**), and Pre-Cx (**D** and **E**). Scale bars = 50 μm. Underlying data can be found in S1 Data. DP, dermal papilla; n.s., not significant; Pre-Cx, pre-cortex.
(TIF)

S11 Fig Macroscopic examples of human hair follicles treated with WAY-316606 (6 days) for hair cycle quantification. **(A)** Macroscopic images of vehicle control hair follicles. (**B**) Macroscopic images of matched patient hair follicles treated with WAY-316606. Scale bars = 1 mm.
(TIF)

S12 Fig WAY-316606 effect on apoptosis within the human HF. WAY-316606 treatment (6 days) did not significantly alter the apoptotic marker TUNEL when analysed below Auber’s line (**A**), within the DP, (**B**) or the DP stalk (**C**) (*n* = 21 HFs control, 20 HFs WAY-316606; 3 male patient samples). Data are expressed as mean ± SEM. Underlying data can be found in S1 Data. AL, Auber’s line; DP, dermal papilla; HF, hair follicle; n.s., not significant.
(TIF)

S1 Table Molecular effects of CsA within the hair follicle. CsA, Cyclosporine A.
(PDF)

S2 Table Review of anagen prolongation by CsA treatment. CsA, Cyclosporine A.
(PDF)

S3 Table Review of SFRP1 in the hair follicle. SFRP1, secreted frizzled related protein 1.
(PDF)

S1 Data (XLSX)

## Abbreviations

*APOBEC3A*: apolipoprotein B mRNA editing enzyme catalytic subunit 3A
*AXIN2*: axis inhibition protein 2
*BDP1*: B double prime 1, subunit of RNA polymerase III transcription initiation factor IIIB
*CCND2*: cyclin D2
*CLCN5*: chloride voltage-gated channel 5
CsA: Cyclosporine A
*CXCL8*: C-X-C motif chemokine ligand 8
*DAB2*: DAB2, clathrin adaptor protein
DC: differentiating cell
DKK1: Dickkopf-related protein 1
DP: dermal papilla
DPC: dermal papilla cell
*EEA1*: early endosome antigen 1
eHFSC: epithelial HF stem cell
*FDCSP*: follicular dendritic cell secreted protein
fdr: false discovery rate
*FLG2*: filaggrin family member 2
HF: hair follicle
IPA: ingenuity pathway analysis
ISH: in situ hybridisation
IWP-2: inhibitor of Wnt production-2
K85: Keratin 85
*KISS1R*: KISS1 receptor
LEF1: lymphoid enhancer binding factor 1
*MALAT1*: metastasis associated lung adenocarcinoma transcript 1
NFATc: nuclear factor of activated T cells
NGS: normal goat serum
*NLRP2*: NLR family pyrin domain containing 2
OCT: optimal cutting temperature
PCA: principal components analysis
*PTX3*: pentraxin 3
qRT-PCR: quantitative real-time PCR
rhSFRP1: recombinant human SFRP1
*RTCA*: RNA 3′-terminal phosphate cyclase
SFRP1: secreted frizzled related protein 1
*SLFN11*: schlafen family member 11
*SPP1*: secreted phosphoprotein 1
TAC: transient amplifying cell
TBS: Tris-buffered saline
*TET2*: tet methylcytosine dioxygenase 2
*WLS*: Wntless
