# Supplementary figures and images for: Identifying novel strategies for treating human hair loss disorders: Cyclosporine A suppresses the Wnt inhibitor, SFRP1, in the dermal papilla of human scalp hair follicles
Source: PLoS Biol. 2018 May 8;16(5):e2003705. doi: 10.1371/journal.pbio.2003705 (PMC5940179; doi:10.1371/journal.pbio.2003705)

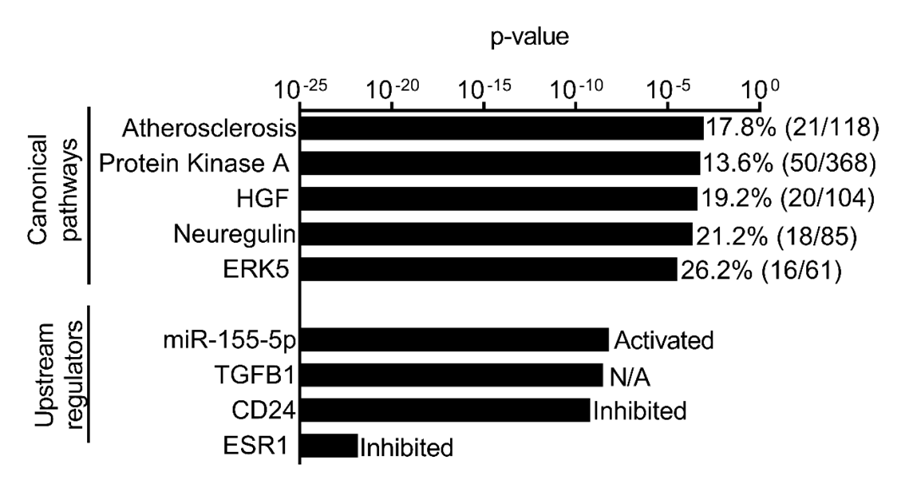

Supplement: S1 Fig — IPA identifies numerous canonical pathways that change with CsA treatment. IPA identifies a significant number of genes changed with CsA treatment that are known to be regulated by certain upstream regulators, predicting whether they are either activated or inhibited. Underlying data can be found in S1 Data. CsA, Cyclosporine A; ERK5, extracellular signal regulated kinase 5; ESR1, estrogen receptor 1; HGF, hepatocyte growth factor; IPA, ingenuity pathway analysis; TGFB1, transforming growth factor beta 1. (TIF) [file pbio.2003705.s001.tif]

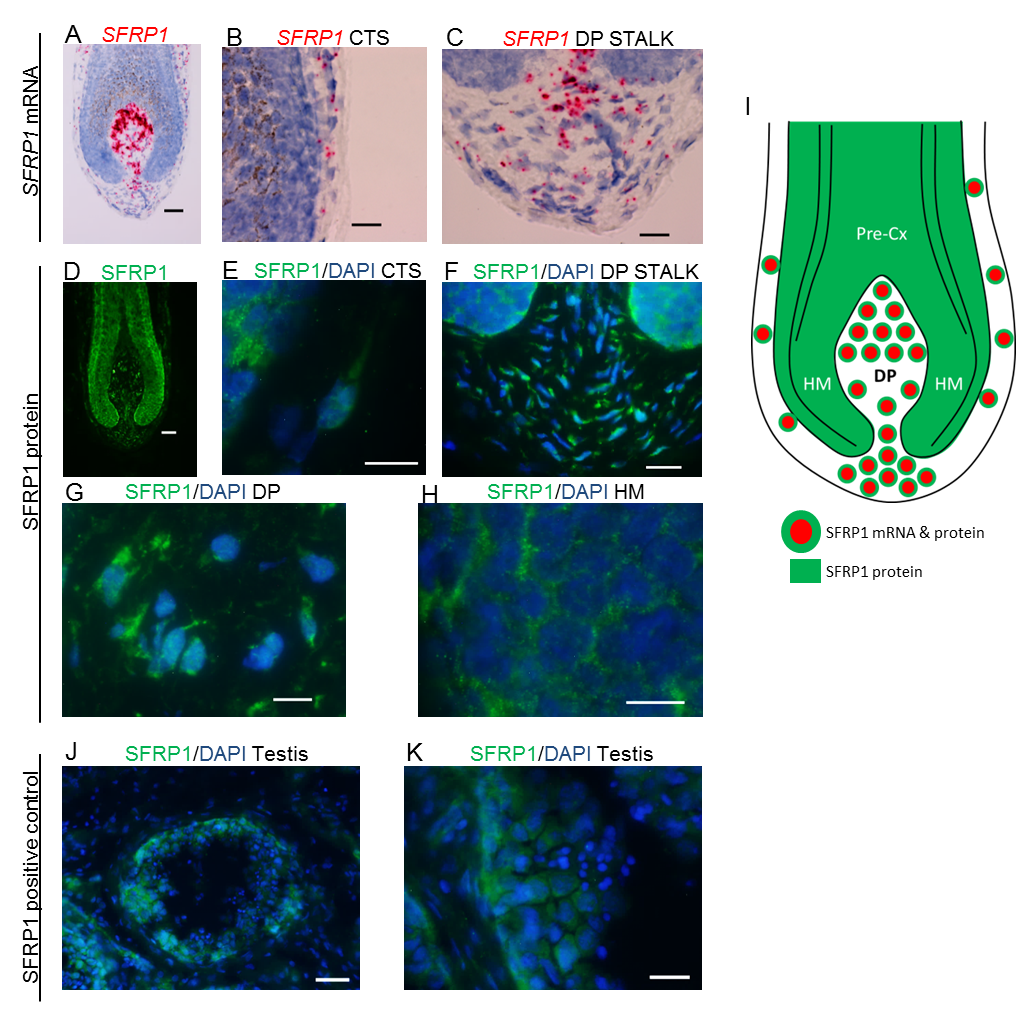

Supplement: S2 Fig — (A–C) Using in situ hybridisation, SFRP1 mRNA can be detected in fibroblast populations within the human HF bulb. (A) SFRP1 mRNA is localised to the DP, (B) CTS, and (C) DP stalk. (D–G) SFRP1 protein can also be visualised in the same population of cells using immunofluorescence. (H) SFRP1 protein is also detected in the adjacent epithelial regions. (I) Diagram of SFRP1 mRNA and protein in the human HF bulb. (J and K) Human testis was used as a positive control for SFRP1 immunofluorescence. Scale bars, A, D, and J = 50 μm; B, C, F, and K = 20 μm; E, G, and H = 10 μm. CTS, connective tissue sheath; DP, dermal papilla; HF, hair follicle; HM, hair matrix; Pre-Cx, pre-cortex; SFRP1, secreted frizzled related protein 1. (TIF) [file pbio.2003705.s002.tif]

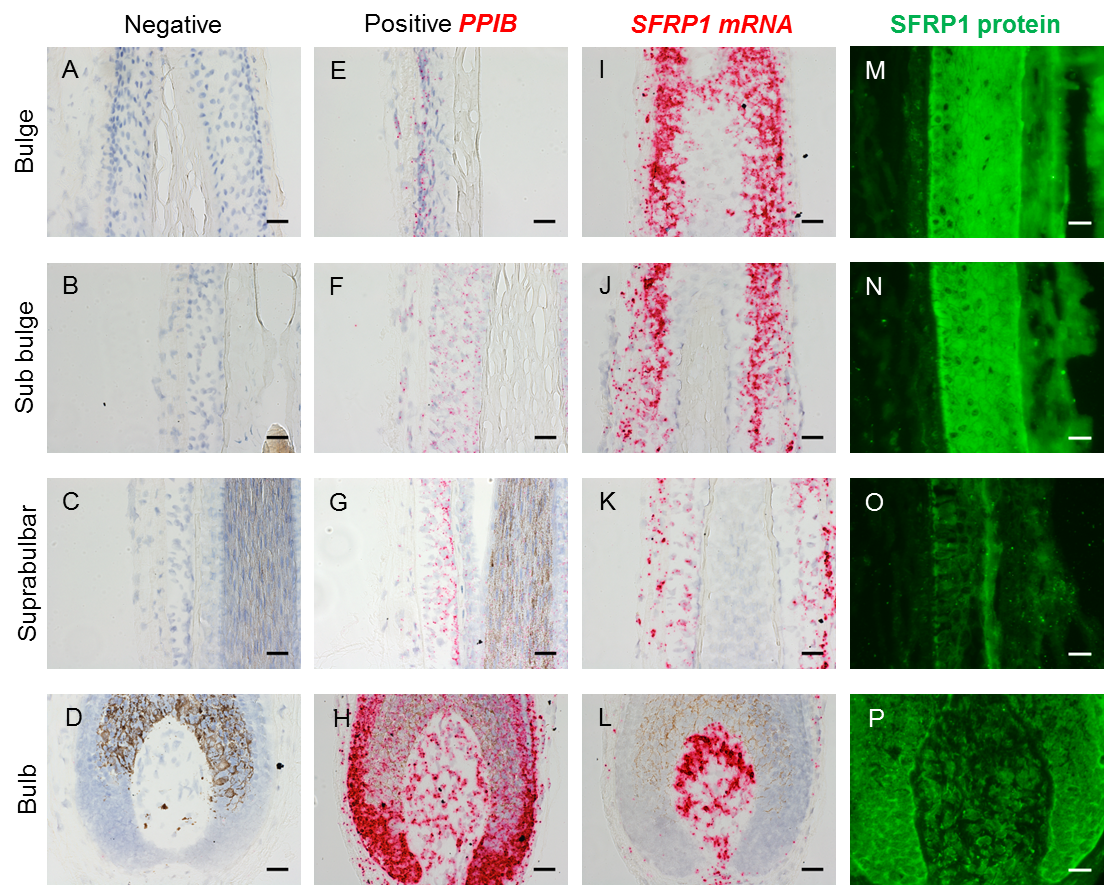

Supplement: S3 Fig — (A–D) negative control for ISH, (E–H) positive control (PPIB mRNA) for ISH, (I–L) SFRP1 mRNA, and (M–P) SFRP1 protein. Scale bars, A–L = 30 μm and M–P = 20 μm. ISH, in situ hybridisation; PPIB, peptidylprolyl isomerase B; SFRP1, secreted frizzled related protein 1. (TIF) [file pbio.2003705.s003.tif]

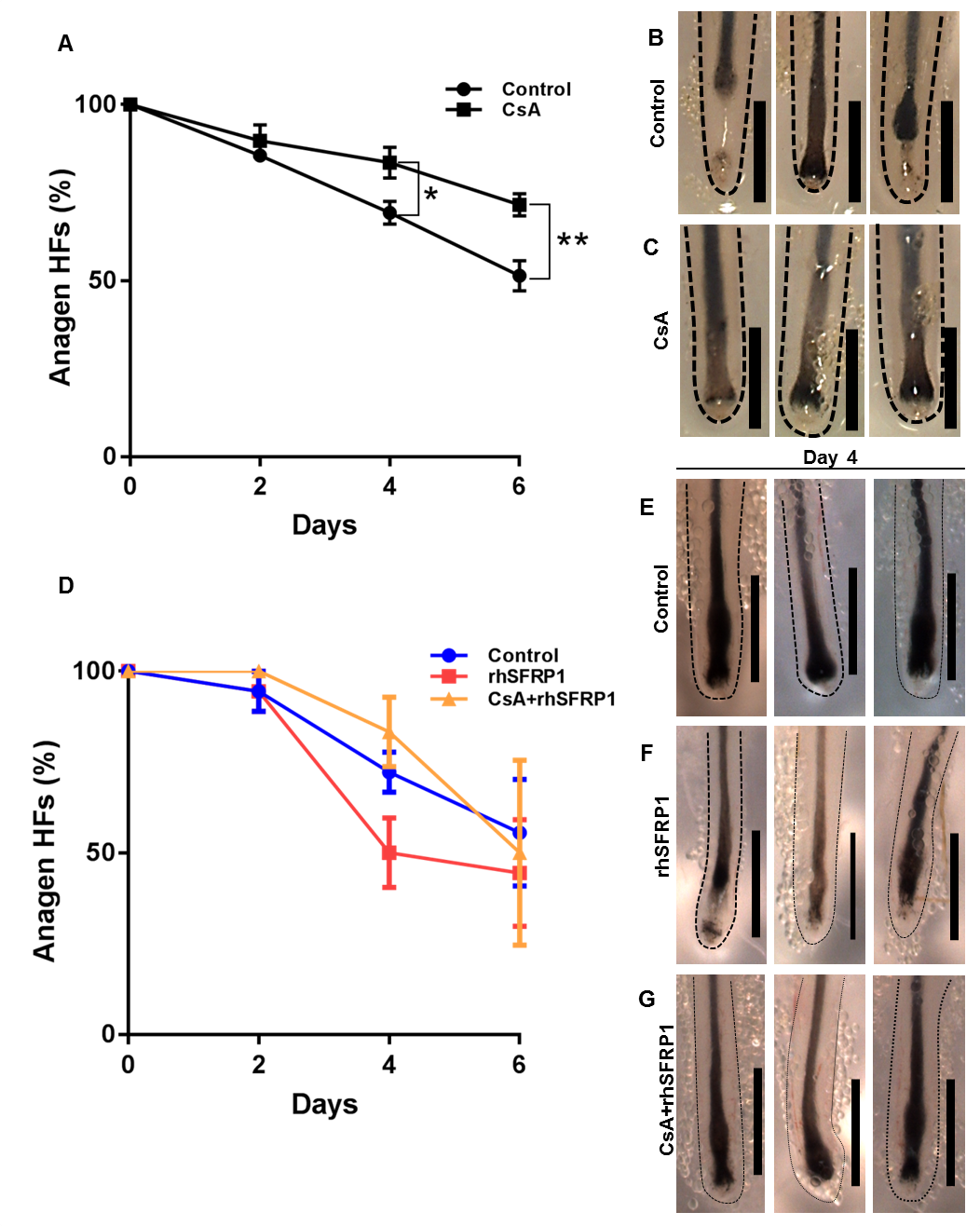

Supplement: S4 Fig — (A) Quantification of hair cycle stage with human HFs (ex vivo) treated with vehicle control and CsA (n = 8–18 HFs per group; from 4 male patient samples). Macroscopic examples of (B) vehicle control HFs and (C) CsA-treated HFs after 6 days in culture. (D) Quantification of hair cycle stage with human HFs (ex vivo) treated with vehicle control, rhSFRP1 alone, and rhSFRP1 with CsA over 6 days (n = 18 HFs per group; from 3 male patient samples). (E) Macroscopic examples of vehicle control HFs, (F) rhSFRP1-treated HFs, and (G) rhSFRP1 plus CsA-treated HFs at day 4. Data are expressed as mean ± SEM; (A) two-tailed unpaired t test; *p < 0.05 and **p < 0.01. Scale bars = 1 mm. Underlying data can be found in S1 Data. CsA, Cyclosporine A; HF, hair follicle; rhSFRP1, recombinant human SFRP1. (TIF) [file pbio.2003705.s004.tif]

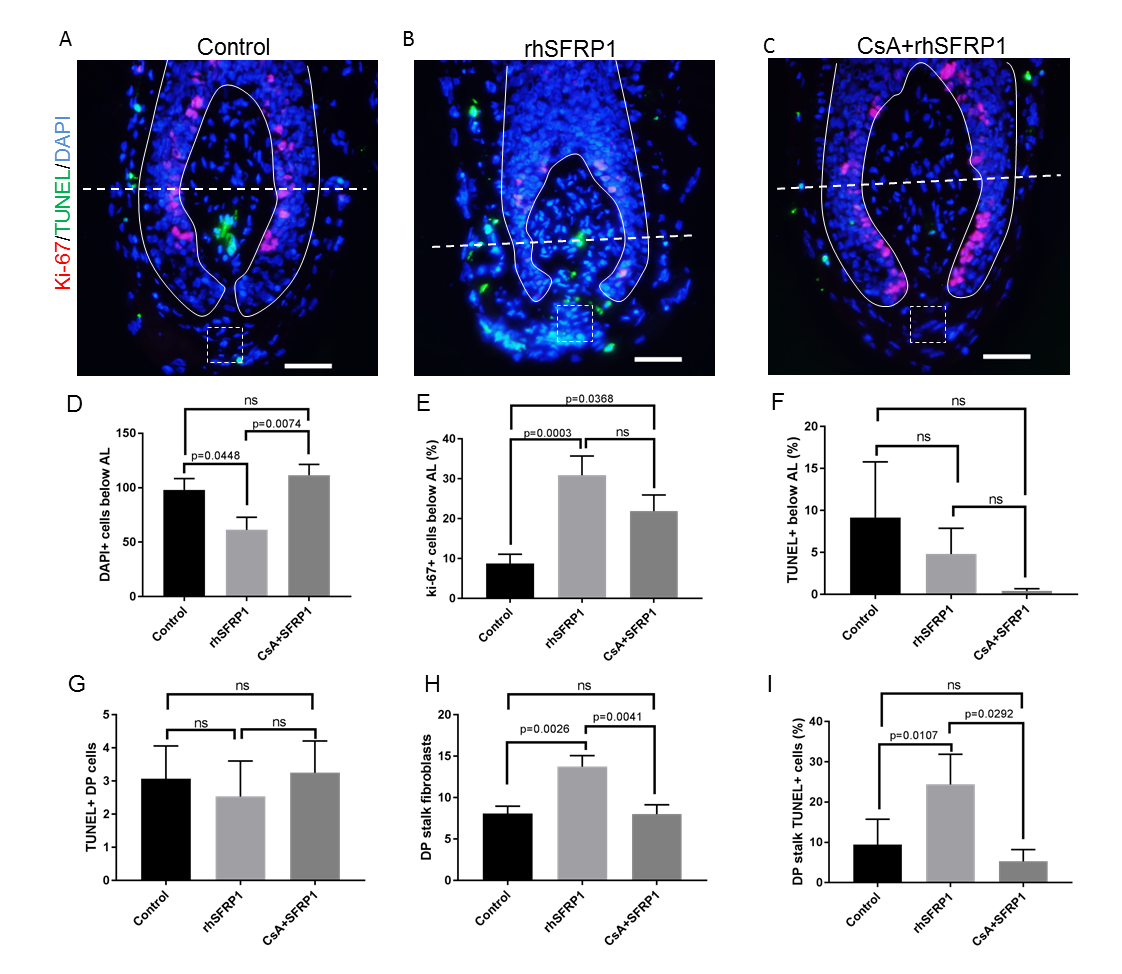

Supplement: S5 Fig — Human HFs were treated with vehicle control (A), rhSFRP1 only (B), or rhSFRP1 with CsA (C) for 6 days and subjected to Ki-67/TUNEL analysis (D–I) (n = 12–15 HFs per group; from 3 male patient samples). D and H = one-way ANOVA; E, F, G, and I = Kruskal-Wallis test; data are expressed as mean ± SEM; dotted white line depicts Auber’s line; scale bars = 50 μm. Underlying data can be found in S1 Data. CsA, Cyclosporine A; HF, hair follicle; rhSFRP1, recombinant human SFRP1. (TIF) [file pbio.2003705.s005.tif]

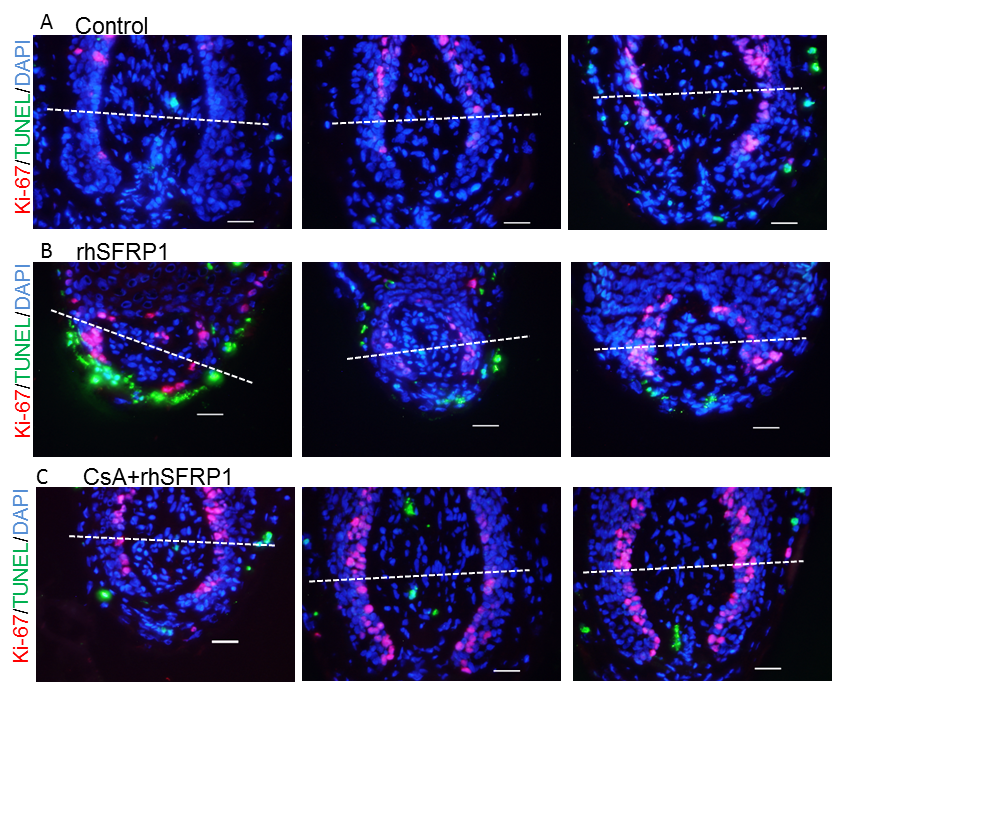

Supplement: S6 Fig — Human HFs were treated with vehicle control (A), rhSFRP1 only (B), or rhSFRP1 with CsA (C) for 6 days and subjected to Ki-67/TUNEL analysis. Dotted white line depicts Auber’s line; scale bars = 30 μm. CsA, Cyclosporine A; HF, hair follicle; rhSFRP1, recombinant human SFRP1. (TIF) [file pbio.2003705.s006.tif]

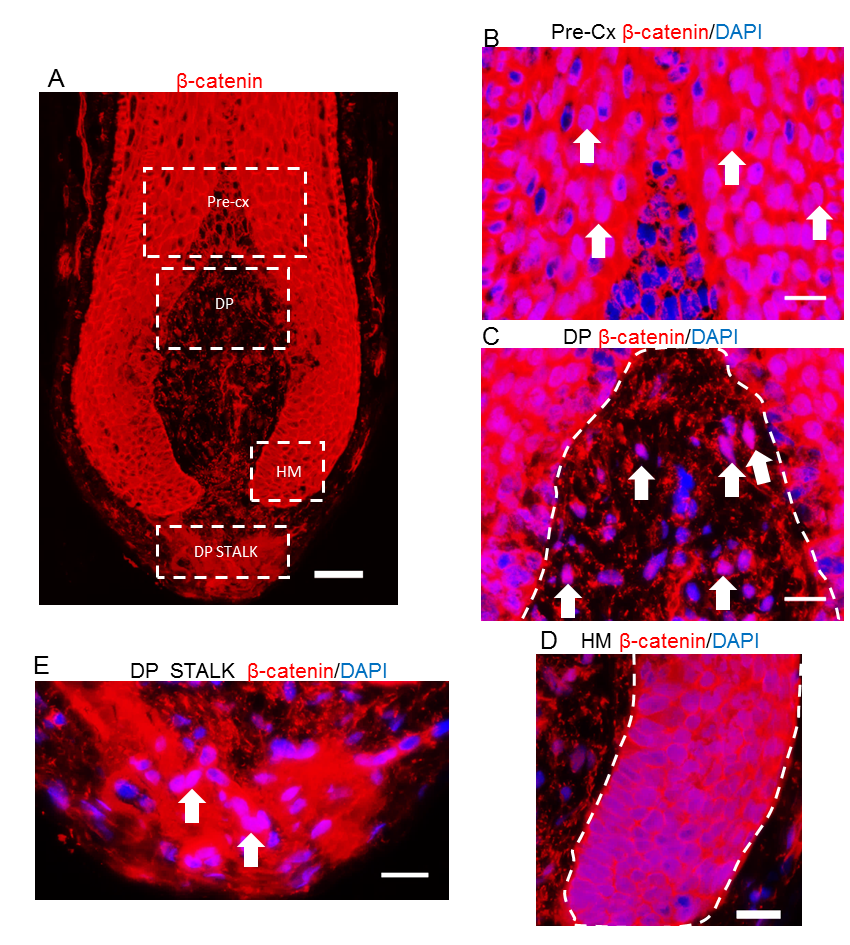

Supplement: S7 Fig — (A–E) Using immunofluorescence, active (nuclear) β-catenin can be detected throughout the human hair follicle bulb, (B) pre-cortex, (C) dermal papilla, (D) hair matrix, and (E) dermal papilla stalk. White arrows depict nuclear β-catenin. Dashed white lines highlight regions of interest. Scale bars, A = 50 μm; B–E = 20 μm. DP, dermal papilla; HM, hair matrix; Pre-Cx, pre-cortex. (TIF) [file pbio.2003705.s007.tif]

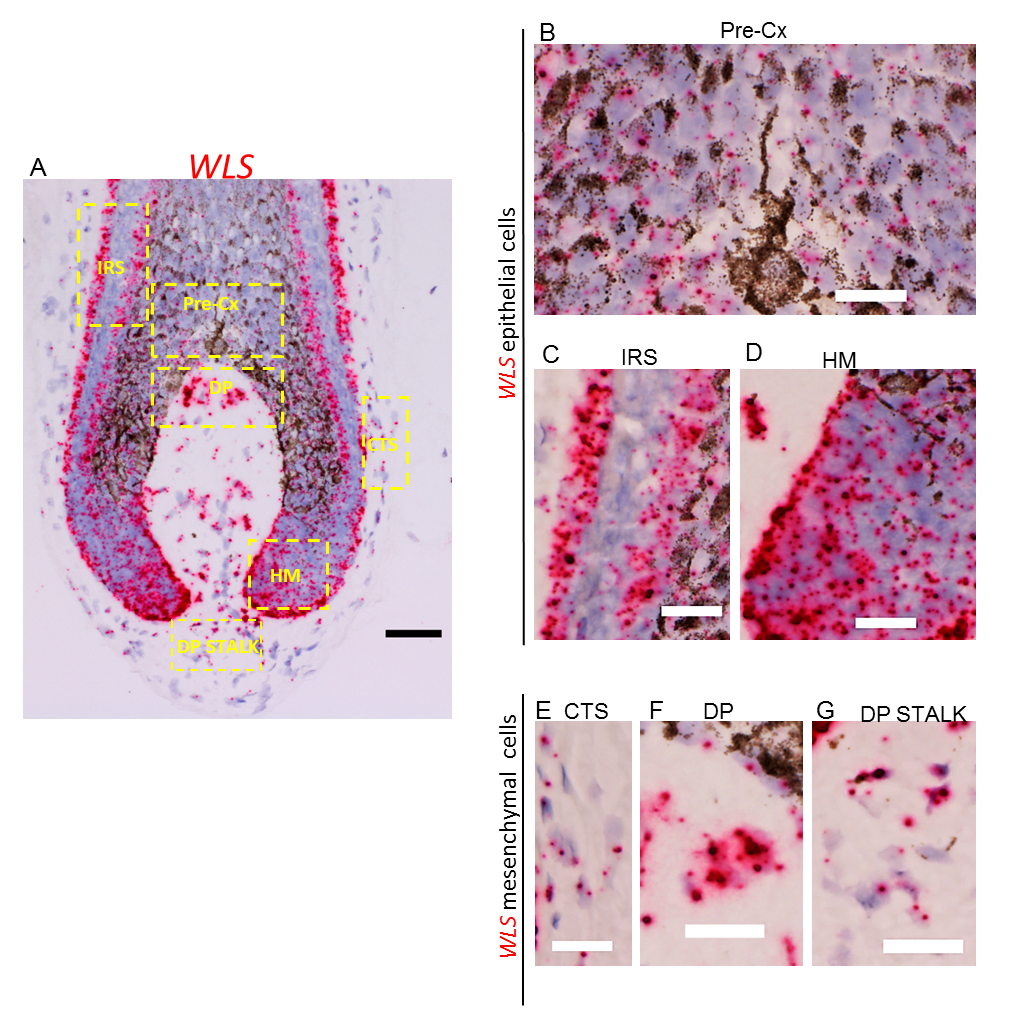

Supplement: S8 Fig — Using in situ hybridisation, WLS mRNA can be detected in both epithelial (B–D) and mesenchymal (E–G) cell populations within the human hair follicle bulb. Dashed yellow lines highlight regions of interest. Scale bars, A = 50 μm, B–G = 20 μm. CTS, connective tissue sheath; DP, dermal papilla; HM, hair matrix; IRS, inner root sheath; Pre-Cx, pre-cortex; WLS, Wntless. (TIF) [file pbio.2003705.s008.tif]

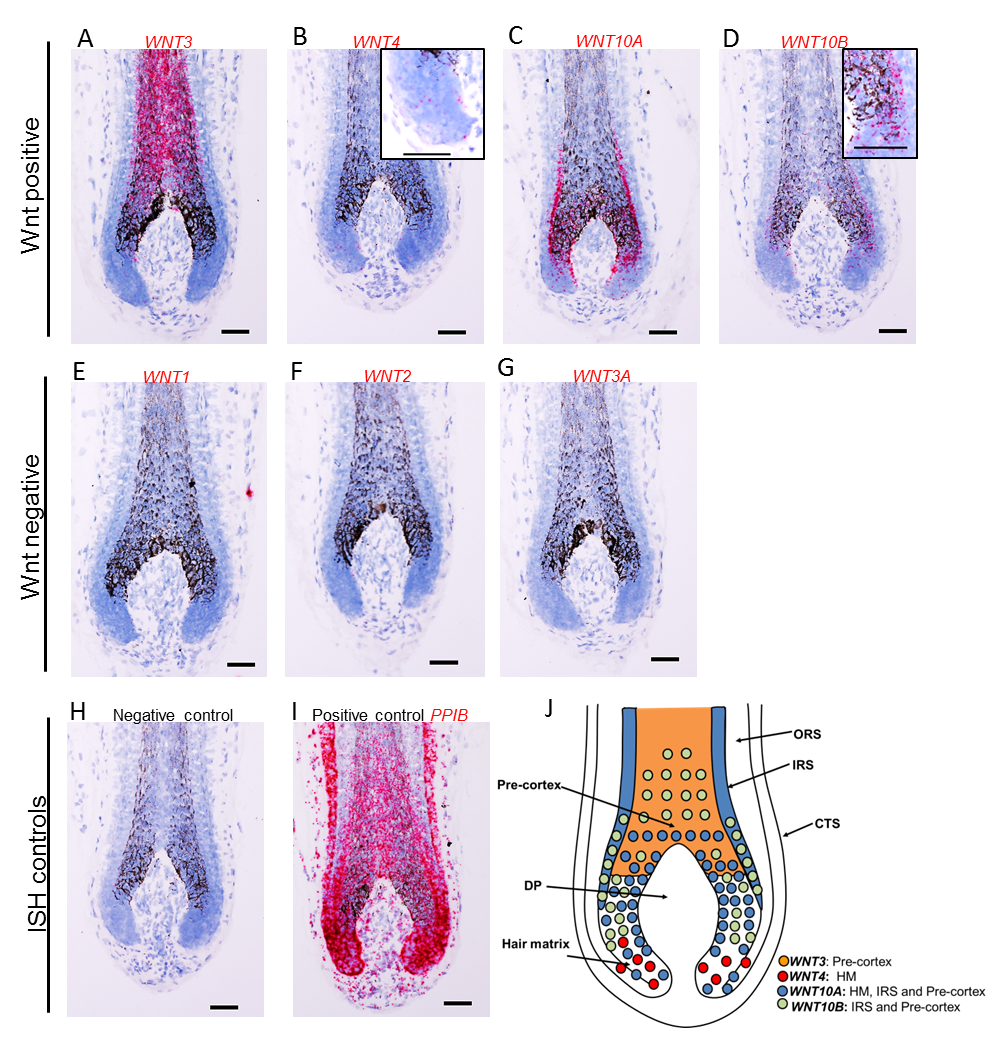

Supplement: S9 Fig — Using ISH, the Wnt ligands WNT3 (A), WNT4 (B), WNT10A (C), and WNT10B (D) can be detected in epithelial cells of the human hair follicle bulb, whereas WNT1 (E), WNT2 (F), and WNT3A (G) were not detected. (H) Negative control and (I) positive control for ISH. (J) Schematic of the Wnt ligands WNT3, WNT4, WNT10A, and WNT10B. Scale bars = 50 μm. CTS, connective tissue sheath; DP, dermal papilla; HM, hair matrix; IRS, inner root sheath; ISH, in situ hybridisation; mRNA, messenger ribonucleic acid; ORS, outer root sheath. (TIF) [file pbio.2003705.s009.tif]

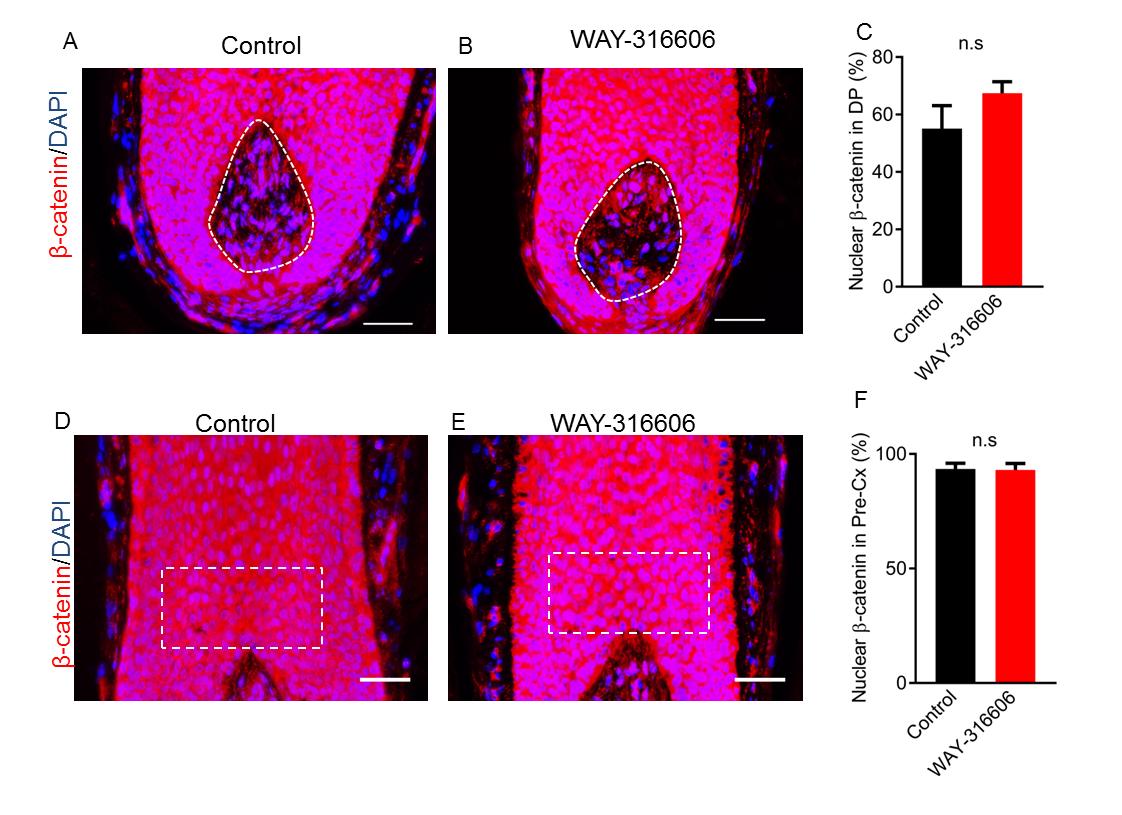

Supplement: S10 Fig — (A–C) Nuclear β-catenin quantification using immunofluorescence within the DP after WAY-316606 treatment (48 hours). (D–F) Nuclear β-catenin quantification using immunofluorescence within the Pre-Cx after WAY-316606 treatment (48 hours) (n = 13 HFs control, 14 HFs WAY-316606; from 3 male patient samples). Data are expressed as mean ± SEM. Dotted lines depict regions analysed, DP (A and B), and Pre-Cx (D and E). Scale bars = 50 μm. Underlying data can be found in S1 Data. DP, dermal papilla; n.s., not significant; Pre-Cx, pre-cortex. (TIF) [file pbio.2003705.s010.tif]

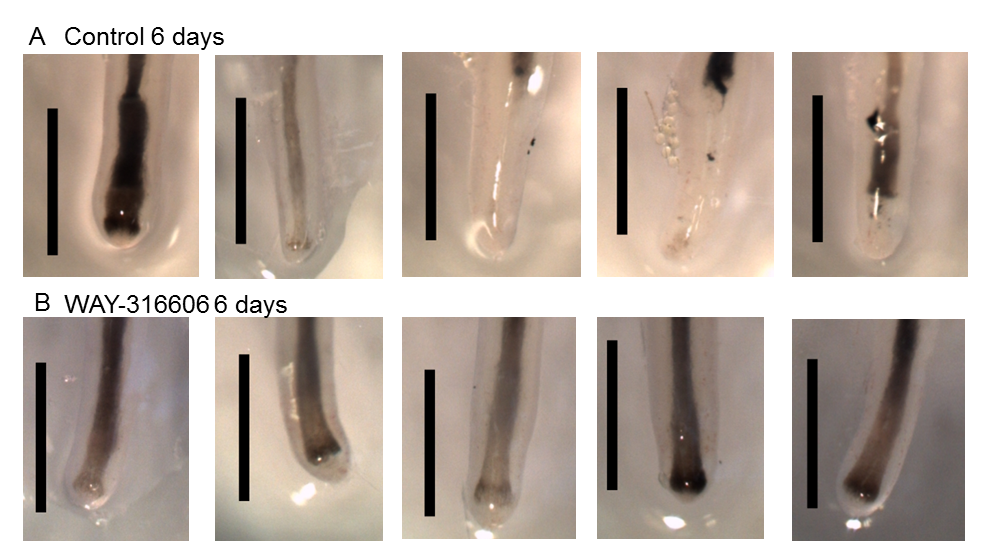

Supplement: S11 Fig — (A) Macroscopic images of vehicle control hair follicles. (B) Macroscopic images of matched patient hair follicles treated with WAY-316606. Scale bars = 1 mm. (TIF) [file pbio.2003705.s011.tif]

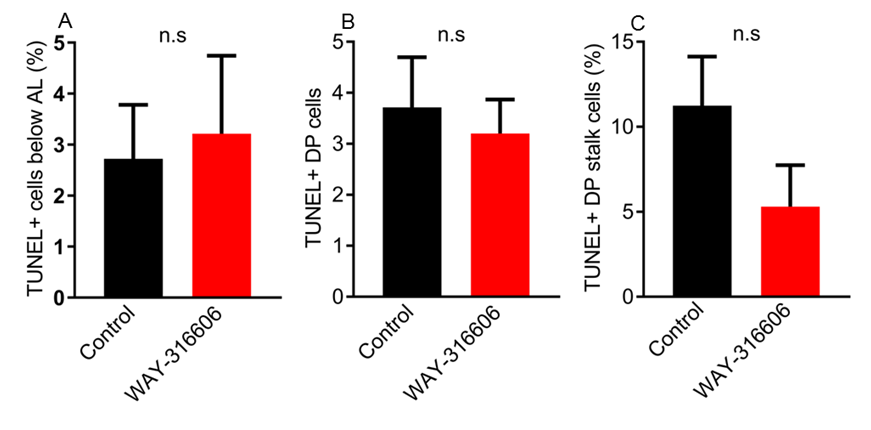

Supplement: S12 Fig — WAY-316606 treatment (6 days) did not significantly alter the apoptotic marker TUNEL when analysed below Auber’s line (A), within the DP, (B) or the DP stalk (C) (n = 21 HFs control, 20 HFs WAY-316606; 3 male patient samples). Data are expressed as mean ± SEM. Underlying data can be found in S1 Data. AL, Auber’s line; DP, dermal papilla; HF, hair follicle; n.s., not significant. (TIF) [file pbio.2003705.s012.tif]
