# Supplementary material for: Identifying novel strategies for treating human hair loss disorders: Cyclosporine A suppresses the Wnt inhibitor, SFRP1, in the dermal papilla of human scalp hair follicles
Source: PLoS Biol. 2018 May 8;16(5):e2003705. doi: 10.1371/journal.pbio.2003705 (PMC5940179; doi:10.1371/journal.pbio.2003705)
Supplement: S1 Table — CsA, Cyclosporine A. (PDF) [file pbio.2003705.s013.pdf]

S1 Table. Molecular effects of cyclosporine A (CsA) within the hair follicle.

CsA biological effect	Model	Changes in mRNA expression	Changes in protein expression	Changes in WNT pathway? (increase/ decrease)	Changes in SFRP1?	Ref
**1. Induction of hair growth**	Sprague-Dawley rat	Amelogenin (↑)<br>Lamp-1 (↓)<br>Lamp-2 (↓)	Amelogenin (↑)<br>Lamp-1 (↓)	✘	✘	1
**1. Increased HF size and density**<br>**2. Delay catagen entry**<br>**3. Prevent AIF release from mitochondria and antagonise CypA**	C57BL/6 mice	n/a	AIF (↓)<br>Hsp70 (↓)<br>Cytochrome c (↓)<br>Calpain1 (↓)<br>Cleaved PARP (↓)<br>CypA (↓)	✘	✘	2
**1.Enhanced hair shaft elongation**<br>**2.Delay catagen**	*Ex vivo* human HF organ culture	*NFATc3* (↑)	n/a	✘	✘	3
**1. Accelerated anagen re-entry**	Transgenic Flash Mice	n/a	n/a	✓<br>Increase	✘	4
**1. Induce transcriptional changes in HFSCs**<br>**2.CsA treated mice develop accelerated hair regrowth during pregnancy**	CD-1 male Mice (microarray)<br><br>CD-1 female Mice (pregnancy experiments)	Microarray data set available<br>*Nfatc1* (↓)<br>*Prlr* (↓)<br>*Pthlh* (↓)<br>*Cd44* (↓)	PRLR (↓)	✘	✘	5
**1.Enhances hair shaft elongation**<br>**2.Increased growth factor expression**	Vibrissae organ culture	*Vegf* (↑)<br>*Hgf* (↑)<br>*Ngf* (↑)<br>*Fst* (↓)	n/a	✘	✘	6
**1.Inhibits NFATc2 nuclear translocation**<br>**2.Reduces cyclin G2 expression in follicular keratinocytes**	Wistar rats	n/a	CCNG2 (↓)	✘	✘	7
**1. Anagen induction**	C3H mice	Microarray data set available	n/a	✓<br>Lef1 mRNA increase within microarray data set	✘	8
**1.Enhances proliferation of ORSK and DPCs**	Human folliculoid microspheres	TGFβ2 (↓)	n/a	✘	✘	9

**within microspheres**	(HFMs)					
**1. Precocious entry into anagen**<br>**2.Increased HFSC colony formation**<br>**3.Inhibits NFATc1 nuclear translocation**	*NFATc1*fl/fl, *K14*-Cre negative<br><br>K14-H2BGFP mice (colony forming experiment)	n/a	NFATc1 (↓)	✘	✘	10
**1. Anagen induction**	C57BL/6 mice<br><br>Cultured Human ORS and DP cells	SAP102 (↑)<br>Erbb2ip (↑)<br>HNF-6 (↓)	n/a	✘	✘	11
**1. Delays catagen**<br>**2. Stimulation of hair growth**	C57BL/6 mice (catagen delay)<br><br>Nude mice (anagen induction)<br><br>Hair epithelial cells	n/a	IL-1α (↑) **note; only in nude mice**<br>TGFβ(↓)<br>p21waf1/cip1 (↓)<br>p27kip1(↓)<br>Involucrin (↓)<br>Loricrin (↓)<br>Bax (↓)<br>P53 (↓)<br>ICE (↓)	✘	✘	12
**1.Promotes hair epithelial proliferation**	C3H/HeNCrj isolated hair epithelial cells	n/a	PKC-α, (↓)<br>PKC-βI (↓)<br>PKC-βII (↓)<br>PKC-η (↓)	✘	✘	13

1. Yoo H-I, Lee G-H, Lee S-Y, et al. Expression of amelogenin and effects of cyclosporin A in developing hair follicles in rats. *J Anat*. 2016;228(1):153-161.

2. Lan S, Liu F, Zhao G, et al. Cyclosporine A increases hair follicle growth by suppressing apoptosis-inducing factor nuclear translocation: A new mechanism. *Fundam Clin Pharmacol*. 2015;29(2):191-203.

3. Hawkshaw NJ, Haslam IS, Ansell DM, Shamalak A, Paus R. Re-evaluating Cyclosporine A as a hair growth-promoting agent in human scalp hair follicles. *J Invest Dermatol*. 2015;135(8):2129-2132.

4. Hodgson SS, Neufeld Z, Villani RM, Roy E, Khosrotehrani K. Transgenic flash mice for in vivo quantitative monitoring of canonical Wnt signaling to track hair follicle cycle dynamics. *J Invest Dermatol*. 2014;134(6):1519-1526.

5. Goldstein J, Fletcher S, Roth E, Wu C, Chun A, Horsley V. Calcineurin/Nfatc1 signaling links skin stem cell quiescence to hormonal signaling during pregnancy and lactation. *Genes Dev*. 2014;28(9):983-994.

6. Xu W, Fan W, Yao K. Cyclosporine A stimulated hair growth from mouse vibrissae follicles in an organ culture model. *J Biomed Res*. 2012;26(5):372-380.

7. Fujimura A, Michiue H, Nishiki T ichi, et al. Expression of a constitutively active calcineurin

encoded by an intron-retaining mRNA in follicular keratinocytes. *PLoS One*. 2011;6(3):e17685.

8. Ishimatsu-Tsuji Y, Soma T, Kishimoto J. Identification of novel hair-growth inducers by means of connectivity mapping. *FASEB J*. 2010;24(5):1489-1496.

9. Havlickova B, Bíró T, Mescalchin A, et al. A human folliculoid microsphere assay for exploring epithelial-mesenchymal interactions in the human hair follicle. *J Invest Dermatol*. 2009;129(4):972-983.

10. Horsley V, Aliprantis AO, Polak L, Glimcher LH, Fuchs E. NFATc1 balances quiescence and proliferation of skin stem cells. *Cell*. 2008;132(2):299-310.

11. Kim CD, Lee M-H, Sohn K-C, et al. Induction of synapse associated protein 102 expression in cyclosporin A-stimulated hair growth. *Exp Dermatol*. 2008;17(8):693-699.

12. Gafter-Gvili A, Sredni B, Gal R, Gafter U, Kalechman Y. Cyclosporin A-induced hair growth in mice is associated with inhibition of calcineurin-dependent activation of NFAT in follicular keratinocytes. *Am J Physiol Cell Physiol*. 2003;284(6):C1593-603.

13. Takahashi T, Kamimura A. Cyclosporin A promotes hair epithelial cell proliferation and modulates protein kinase C expression and translocation in hair epithelial cells. *J Invest Dermatol*. 2001;117(3):605-611.
