# Supplementary material for: Identifying novel strategies for treating human hair loss disorders: Cyclosporine A suppresses the Wnt inhibitor, SFRP1, in the dermal papilla of human scalp hair follicles
Source: PLoS Biol. 2018 May 8;16(5):e2003705. doi: 10.1371/journal.pbio.2003705 (PMC5940179; doi:10.1371/journal.pbio.2003705)
Supplement: S2 Table — CsA, Cyclosporine A. (PDF) [file pbio.2003705.s014.pdf]

S2 Table. Review of anagen prolongation by CsA treatment

Model	CsA administration	Species	Notes	Reference
Ex vivo (isolated HFs)	Culture medium	Human	CsA at 10^{-7}M concentration gave a 42% greater mean hair shaft elongation after 15 days of treatment. 43% of CsA treated HFs were still growing at day 15 compared to the 2% of control HFs.	1
Ex vivo (isolated HFs)	Culture medium	Human	CsA treated HFs inhibited catagen morphology after 6 days of treatment (10^{-7}M concentration).	2
In vivo (xenotransplant)	Topical	Human HFs grafted onto nude mice	CsA treatment prolongs anagen.	3
In vitro (isolated hair follicle epithelial cells)	Culture medium	Male nude mice of Balb/c origin and<br>Female C57BL/6 mice	Dose dependent reduction of early and late apoptosis.	4
In vivo	Intraperitoneal	C57BL/6 mice	85% of control mice entered telogen via catagen on day 19 whereas only 6% of CsA treated mice entered telogen.	5
In vivo	Topical	6-9 week old female C57BL/6 mice	CsA inhibited DEX induced catagen. The activity of CsA was concentration dependent.	6
In vivo	Intraperitoneal	6-8 week old female C57BL/6 mice	CsA inhibited DEX induced catagen.	7
In vivo-(chemotherapy induced alopecia)	Intraperitoneal	6-8 week old female C57BL/6 mice	CsA retard cyclophosphamide-induced alopecia.	8
Ex vivo (vibrissae HFs)	Culture medium	Male and Female C57BL/6 mice	CsA prevents catagen morphology.<br>63% of control HFs ejected the hair shaft on day 10 while CsA treatment only had 23%. CsA	9

			treated HFs had significantly higher Ki-67+ cells compared to control. There were more TUNEL+ cells in control that CsA treated HFs.	
In vivo	Oral	Nude mice	CsA inhibited catagen in a dose dependent manner. CsA altered cytokine and apoptotic protein expression.	10
In vivo	Intraperitoneal	28 day old Male nude mice	CsA enhanced proliferation and inhibited terminal differentiation.	11

1. Taylor M, Ashcroft ATT, Messenger AG. Cyclosporin A prolongs human hair growth in vitro. *J Invest Dermatol*. 1993;100(3):237-239.
2. Hawkshaw NJ, Haslam IS, Ansell DM, Shamalak A, Paus R. Re-evaluating Cyclosporine A as a hair growth-promoting agent in human scalp hair follicles. *J Invest Dermatol*. 2015;135(8):2129-2132.
3. Gilhar A, Pillar T, Etzioni A. The effect of topical cyclosporin on the immediate shedding of human scalp hair grafted onto nude mice. *Br J Dermatol*. 1988;119(6):767-770.
4. Gafter-Gvili A, Sredni B, Gal R, Gafter U, Kalechman Y. Cyclosporin A-induced hair growth in mice is associated with inhibition of calcineurin-dependent activation of NFAT in follicular keratinocytes. *Am J Physiol Cell Physiol*. 2003;284(6):C1593-603.
5. Paus R, Henz B, Maurer M. Hair growth control by immunosuppression. *Arch Dermatol Res*. 1996;288(7):408-410.
6. Maurer M, Handjiski B, Paus R. Hair growth modulation by topical immunophilin ligands: induction of anagen, inhibition of massive catagen development, and relative protection from chemotherapy-induced alopecia. *Am J Pathol*. 1997;150(4):1433-1441.
7. Paus R, Handjiski B, Czarnetzki BM, Eichmuller S. A murine model for inducing and manipulating hair follicle regression (catagen): effects of dexamethasone and cyclosporin A. *J Invest Dermatol*. 1994;103(2):143-147.
8. Paus R, Handjiski B, Eichmüller S, Czarnetzki BM. Chemotherapy-induced alopecia in mice. Induction by cyclophosphamide, inhibition by cyclosporine A, and modulation by dexamethasone. *Am J Pathol*. 1994;144(4):719-734.
9. Xu W, Fan W, Yao K. Cyclosporine A stimulated hair growth from mouse vibrissae follicles in an organ culture model. *J Biomed Res*. 2012;26(5):372-380.
10. Gafter-Gvili A, Kalechman Y, Sredni B, Gal R, Gafter U. Cyclosporin A-induced hair growth in mice is associated with inhibition of hair follicle regression. *Arch Dermatol Res*. 2004;296(6):265-269.
11. Fujimura A, Michiue H, Nishiki T ichi, et al. Expression of a constitutively active calcineurin encoded by an intron-retaining mRNA in follicular keratinocytes. *PLoS One*. 2011;6(3):e17685.
