# Supplementary material for: Identifying novel strategies for treating human hair loss disorders: Cyclosporine A suppresses the Wnt inhibitor, SFRP1, in the dermal papilla of human scalp hair follicles
Source: PLoS Biol. 2018 May 8;16(5):e2003705. doi: 10.1371/journal.pbio.2003705 (PMC5940179; doi:10.1371/journal.pbio.2003705)
Supplement: S3 Table — SFRP1, secreted frizzled related protein 1. (PDF) [file pbio.2003705.s015.pdf]

S3 Table. Review of SFRP1 in the hair follicle.

Model	SFRP1 mRNA/protein	SFRP1 location	SFRP1 biological effect?	Changes in β-catenin signalling by SFRP1?	Ref
**Human frozen skin**	mRNA	Melanocyte precursors within bulge region	n/a	✘	1
**Murine**	mRNA	Anagen and telogen HFSCs	n/a	✘	2
**Murine; K14-H2BGFP Lef1-RFP Sox2GFP K14-RFP Sox9-GFP Crabp1-GFP**	mRNA	ORS, Mx, DP, HFSCs	n/a	✘	3
**C57BL/6 skin**	mRNA	n/a	n/a	✘	4
**C57BL/6 neonatal**	mRNA	Dermal cells	Impaired HF formation	✘	5
**BALB/C mice**	mRNA	n/a	n/a	✘	6
**Human isolated DP**	mRNA	DP	n/a	✘	7
**HrHp/HrHp mice**	mRNA	n/a	n/a	✘	8
**HrHp/HrHp mice**	mRNA	n/a	n/a	✘	9
**Sox2eGFP mice**	mRNA	Dermal papilla populations GFP-CD133- GFP-CD133+ GFP+ CD133+	n/a	✘	10
**Human frozen skin**	mRNA	HFSCs	n/a	✘	11
**Immortalized balding and non-balding DP cells**	mRNA	DP cells	n/a	✘	12

1. Goldstein NB, Koster MI, Hoaglin LG, et al. Isolating RNA from precursor and mature melanocytes from human vitiligo and normal skin using laser capture microdissection. *Exp Dermatol*. 2016;25(10):805-811.

2. Lim X, Tan SH, Yu K Lou, Lim SBH, Nusse R. Axin2 marks quiescent hair follicle bulge stem cells that are maintained by autocrine Wnt/β-catenin signaling. *Proc Natl Acad Sci*. 2016;113(11):E1498-E1505.

3. Rezza A, Wang Z, Sennett R, et al. Signaling networks among stem cell precursors, transit-amplifying progenitors, and their niche in developing hair follicles. *Cell Rep*. 2016;14(12):3001-3018.

4. Lee J, Lee K, Chung H. Investigation of transcriptional gene profiling in normal murine hair follicular substructures using next-generation sequencing to provide potential insights into skin disease. *Cell Transpl*. 2016;25(2):377-399.

5. Bak S-S, Kim MK, Kim JC, Sung YK. Follistatin and secreted frizzled-related protein 1, OVO homolog-like 1-regulated genes, are important for hair follicle neogenesis. *Exp Dermatol*. 2015;24(7):550-551.

6. Kim BK, Yoon SK. Expression of Sfrp2 is increased in catagen of hair follicles and inhibits keratinocyte proliferation. *Ann Dermatol*. 2014;26(1):79-87.

7. Higgins CA, Chen JC, Cerise JE, Jahoda CAB, Christiano AM. Microenvironmental reprogramming by three-dimensional culture enables dermal papilla cells to induce de novo human hair-follicle growth. *Proc Natl Acad Sci U S A*. 2013;110(49):19679-19688.

8. Choi JH, Kim BK, Kim JK, Lee HY, Park J, Yoon SK. Downregulation of Foxe1 by HR suppresses Msx1 expression in the hair follicles of Hr HP mice. *BMB Rep*. 2011;44(7):478-483.

9. Kim B-K, Baek I-C, Lee H-Y, Kim J-K, Song H-H, Yoon SK. Gene expression profile of the skin in the “hairpoor” (HrHp) mice by microarray analysis. *BMC Genomics*. 2010;11:640.

10. Driskell RR, Giangreco A, Jensen KB, Mulder KW, Watt FM. Sox2-positive dermal papilla cells specify hair follicle type in mammalian epidermis. *Development*. 2009;136(16):2815-2823.

11. Ohyama M, Terunuma A, Tock CL, et al. Characterization and isolation of stem cell-enriched human hair follicle bulge cells. *J Clin Invest*. 2006;116(1):249-260.

12. Chew EGY, Tan JHJ, Bahta AW, et al. Differential expression between human dermal papilla cells from balding and non-balding scalps reveals new candidate genes for androgenetic alopecia. *J Invest Dermatol*. 2016;136(8):1559-1567.
